# POU-domain factor Brn3a regulates both distinct and common programs of gene expression in the spinal and trigeminal sensory ganglia

**DOI:** 10.1186/1749-8104-2-3

**Published:** 2007-01-19

**Authors:** S Raisa Eng, Iain M Dykes, Jason Lanier, Natalia Fedtsova, Eric E Turner

**Affiliations:** 1 Department of Psychiatry, University of California, San Diego and VA San Diego Healthcare System, Gilman Drive, La Jolla, CA 92093-0603, USA

## Abstract

**Background:**

General somatic sensation is conveyed to the central nervous system at cranial levels by the trigeminal ganglion (TG), and at spinal levels by the dorsal root ganglia (DRG). Although these ganglia have similar functions, they have distinct embryological origins, in that both contain neurons originating from the neural crest, while only the TG includes cells derived from the placodal ectoderm.

**Results:**

Here we use microarray analysis of E13.5 embryos to demonstrate that the developing DRG and TG have very similar overall patterns of gene expression. In mice lacking the POU-domain transcription factor Brn3a, the DRG and TG exhibit many common changes in gene expression, but a subset of Brn3a target genes show increased expression only in the TG. In the wild-type TG these Brn3a-repressed genes are silent, yet their promoter regions exhibit histone H3-acetylation levels similar to constitutively transcribed gene loci. This increased H3-acetylation is not observed in the DRG, suggesting that chromatin modifications play a role in cell-specific target gene regulation by Brn3a.

**Conclusion:**

These results demonstrate that one developmental role of Brn3a is to repress potential differences in gene expression between sensory neurons generated at different axial levels, and to regulate a convergent program of developmental gene expression, in which functionally similar populations of neurons are generated from different embryological substrates.

## Background

The generation of cellular diversity in the developing vertebrate nervous system is one of the most complex problems in biology, and a large number of transcription factors have been identified that orchestrate neurodevelopment and regulate the molecular identity of neurons. Perhaps the best-studied systems for the establishment of neuronal phenotypes in vertebrates are the primary input (sensory) and output (motor) pathways of the peripheral sensory ganglia, spinal cord, and brainstem. In these areas, several key transcriptional regulators have been identified, many of which are members of the basic-helix-loop-helix (bHLH) and homeodomain transcription factor classes [1,2].

The peripheral sensory nervous system is organized anatomically according to the nature of the external information reported to the central nervous system (CNS). The sensory modalities of pain, touch, temperature and proprioception are transduced by sensory neurons innervating the skin and musculoskeletal structures, and are referred to as general somatic sensation. Peripheral sensory neurons also convey the senses of taste and hearing, and visceral sensation, which reports the state of the internal organs. At spinal levels, general somatic sensation is conveyed by the dorsal root or spinal ganglia (DRG), while in the anterior head and face, these sensory modalities are mediated by the trigeminal ganglion (TG).

Surprisingly, in spite of the similar functions of the DRG and TG, these ganglia have rather different embryological origins. The DRG are formed entirely from spinal neural crest. In contrast, the TG is formed in part from the ophthalmic and maxillo-mandibular placodes originating in the surface ectoderm, as well as cranial neural crest cells derived from rhombomere 2, which migrate and condense to form the ganglion [3,4]. The trigeminal system also includes a population of sensory neurons that reside outside the anatomical ganglion, the mesencephalic trigeminal (mesV), the exact origin of which is still somewhat controversial [5,6]. The developmental mechanisms that lead to the differentiation of functionally similar populations of neurons from these different embryological sources are not well understood.

Transcriptional regulators of sensory development may be broadly divided into early factors that are essential for neurogenesis, pan-sensory factors that begin to be expressed around the time of cell-cycle exit, and late factors that characterize specific sensory subtypes. In mice, the proneural bHLH factors Ngn1 and Ngn2 are expressed transiently from embryonic day 8.5 in cranial sensory precursors and have been shown to have a crucial role in neurogenesis [7-9]. Around the time of ganglion condensation and cell cycle exit, beginning at E9.5–10.5, nearly all sensory neurons at both spinal and cranial levels co-express the homeodomain transcription factors Brn3a and Islet1 [1,10]. Later in development, factors associated with the development of specific sensory subtypes include the runt family factors Runx1 and Runx3, the variant homeodomain protein Prrxl1/DRG11, and the Ets family member Etv1/Er81 [1,11-15].

Studies of Brn3a knockout mice have shown that this factor is required for correct axon growth, target innervation, and survival of TG and DRG neurons [16-19]. Microarray studies of the developing TG of Brn3a null mice have shown that Brn3a is required for a complex program of sensory gene expression [20]. In the present study, we examine the role of Brn3a in sensory neurogenesis at truncal and cranial levels. In normal mice, the developing DRG and TG have very similar patterns of global gene expression. The loss of Brn3a expression in the developing DRG leads to marked changes in the expression of specific neurotransmitters, receptors, developmental regulators, mediators of signal transduction, and transcription factors. Many of these changes are conserved between the TG and DRG of Brn3a knockout mice, but certain transcripts are markedly increased only in the cranial ganglia. The promoter regions of these normally silent but differentially regulated genes are hyperacetylated only in the TG, which may indicate a latent state of 'expressability' that can differ between spinal and cranial levels. Thus, a key developmental role of Brn3a may be to repress potential differences in gene expression between developing DRG and TG neurons, and thus promote the generation of functionally similar populations of neurons from different embryological sources.

## Results

### Global gene expression is highly conserved in sensory neurons from different axial levels

**Figure 1:**
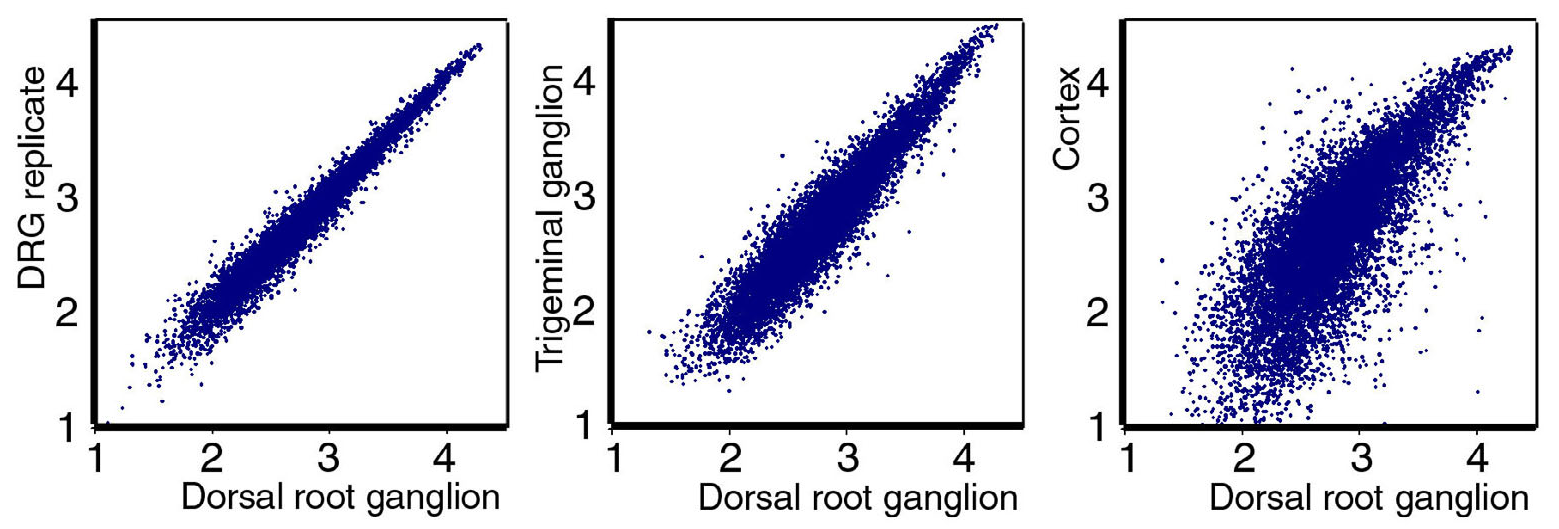
Analysis of global gene expression in embryonic neural tissue. Analysis of global gene expression was performed using E13.5 DRG, E13.5 TG and E16.5 cerebral cortex samples. Only probe sets on the Affymetrix 430A array are shown, and transcripts with 'absent' calls in all three samples were excluded from the analysis. **(a) **Plot of DRG expression versus a replicate DRG assay closely approximates a diagonal line indicating equal expression in the two samples. **(b) **Plot of DRG expression versus the TG indicates very similar global gene expression in the two samples, with few points lying far from the diagonal. **(c) **Plot of DRG expression versus the embryonic cerebral cortex indicates a large number of transcripts that are differentially expressed between the two samples. Axis is graduated in log10 of scaled signal.

To begin to compare the molecular program of sensory neuron development at spinal and cranial levels, we performed global analysis of gene expression in the developing DRG and TG. E13.5 was chosen for this comparison because, at this stage, most of the neurons of the DRG and TG have exited the cell cycle and have begun to express definitive markers of neurogenesis. Because sensory neurons have conserved functions at spinal and cranial levels, we hypothesized that the global pattern of gene expression in the DRG would be much more similar to the TG than to other neural tissues. Figure 1 illustrates a comparison of gene expression in the DRG to a replicate analysis from the same tissue, to the TG, and to the embryonic neocortex. These results show a high degree of overall similarity in gene expression between the sensory ganglia from different axial levels, and confirm that there is much greater divergence between the patterns of gene expression in the DRG and the developing cerebral cortex.

Overall, only a very small fraction of the approximately 44,000 transcripts assayed by the array showed profound differences in expression between the DRG and TG (Table 1). Significantly more transcripts were uniquely expressed in the DRG, and seven out of ten of the transcripts with the highest relative expression in the DRG encoded Hox transcription factors, an expected finding given the axial restriction of Hox expression. Several of the other differentially expressed genes revealed in the microarray analysis could be validated by *in situ *hybridization (see additional file 1), although some differences appear to represent anomalous findings that may be related to factors such as high expression in peripheral glia (Ednrb, Sostdc1).

**Table 1:** Transcripts differentially expressed in the DRG and TG

Transcript	Symbol	Class	Fold
**Greater expression in the DRG**			
Homeobox B3	Hoxb3^2^	TX	(90.4)
Homeobox A9	Hoxa9	TX	(90.2)
Homeobox A5	Hoxa5^2^	TX	(44.2)
Etv1; ER81	Etv1^3^	TX	(40.2)
Homeobox C8	Hoxc8^2^	TX	(38.4)
Homeobox B6	Hoxb6	TX	(27.6)
Hoxd4	Hoxd4	TX	(16.6)
Follistatin	Fst^2^	Dev	(12.9)
Leucine-rich repeat-containing G protein-coupled receptor 6	Lgr6	Other	(11.8)
Receptor (calcitonin) activity modifying protein 2	Ramp2^3^	ST	10.9
Ca^+ ^channel, L type, alpha 1C subunit	Cacna1c	NT	9.2
Homeobox B8	Hoxb8	TX	(9.1)
BTB and CNC homology 2	Bach2	TX	(8.8)
Sclerostin domain containing 1	Sostdc1	Dev	8.5
Laminin, alpha 2	Lama2	Other	(7.3)
**Selected**			
Purkinje cell protein 4	Pcp4	Unk	(5.7)
LIM domain binding 2	Ldb2^2^	TX	5.6
Zinc finger protein of the cerebellum 1	Zic1^2^	TX	5.3
Gap junction membrane channel protein alpha 1	Gja1^2^	NT	5.1
Protein tyrosine phosphatase, receptor type, E	Ptpre^3^	ST	5.0
Early B-cell factor 1; Olf1	Ebf1	TX	5.0
**U74 array**			
Regulator of G-protein signaling 4	RGS4	NT	6.1
Endothelin receptor type B	EDNRB	Other	3.0
Anthrax toxin receptor 2	Antxr2	Unk	3.0
			
**Greater expression in the TG**			
Microfibrillar-associated protein 4	Mfap4	Unk	(20.6)
Lectin, galactose binding, soluble 7	Lgals7	AX	(11.3)
MyoD family inhibitor	Mdfi	TX	(9.6)
Suppressor of cytokine signaling 3	Socs3^2^	ST	(6.4)
RNA imprinted and accumulated in nucleus	Rian	Unk	6.1
Musashi homolog 2	Msi2h	Other	6.0
**U74 array**			
Neuropeptide Y receptor 1	NpyR1	NT	8.6

The principal analysis was conducted with the murine 430 array, with selected results identified using the U74A+B array set added. All listed transcripts showed increased expression (change *p *< 0.005) in two independent replicates. Fold values represent the ratio of the means of two determinations for each tissue. Fold changes in parentheses indicate an absent call, that is, below the statistically reliable limit of detection, in the lower expressing sample. Transcripts with expression levels less than 40% of the scaled mean are excluded. Superscript numerals represent the number of probe sets concordant for changed expression for a given gene. AX, axonogenesis; Dev, development; NT, neurotransmission; ST, signal transduction; Syn, synaptogenesis; TX, transcription; Unk, unknown. Greater expression in the DRG: confirmation of selected results by in situ hybridization and immunofluorescence appears in additional file 1. Differential expression of Ramp2 could not be confirmed by ISH. Different levels of Zic1 and follistatin appear to be due to high expression in the spinal cord adjacent to the DRG, rather than the DRG itself. Sostdc1 and EDNRB showed increased DRG expression by ISH but in a pattern more characteristic of glial than neuronal expression, with signal concentrated in nerve roots. The expression of RGS4 in the developing sensory ganglia has been previously described [43], and its higher expression in the DRG at E13.5 appears to be due to the dynamic developmental regulation of this gene, rather than a persisting difference in expression between the DRG and TG. Greater expression in the TG: *in situ *hybridization confirmed some changes (see additional file 1). However, Mfap4 was widely expressed in embryonic tissues. Socs3 ISH exhibited relatively greater expression in the TG, but overall expression levels were low. Differential expression of the supressor of cytokine signaling Socs3 in the TG and DRG has previously been noted [44].

**Figure 2:**
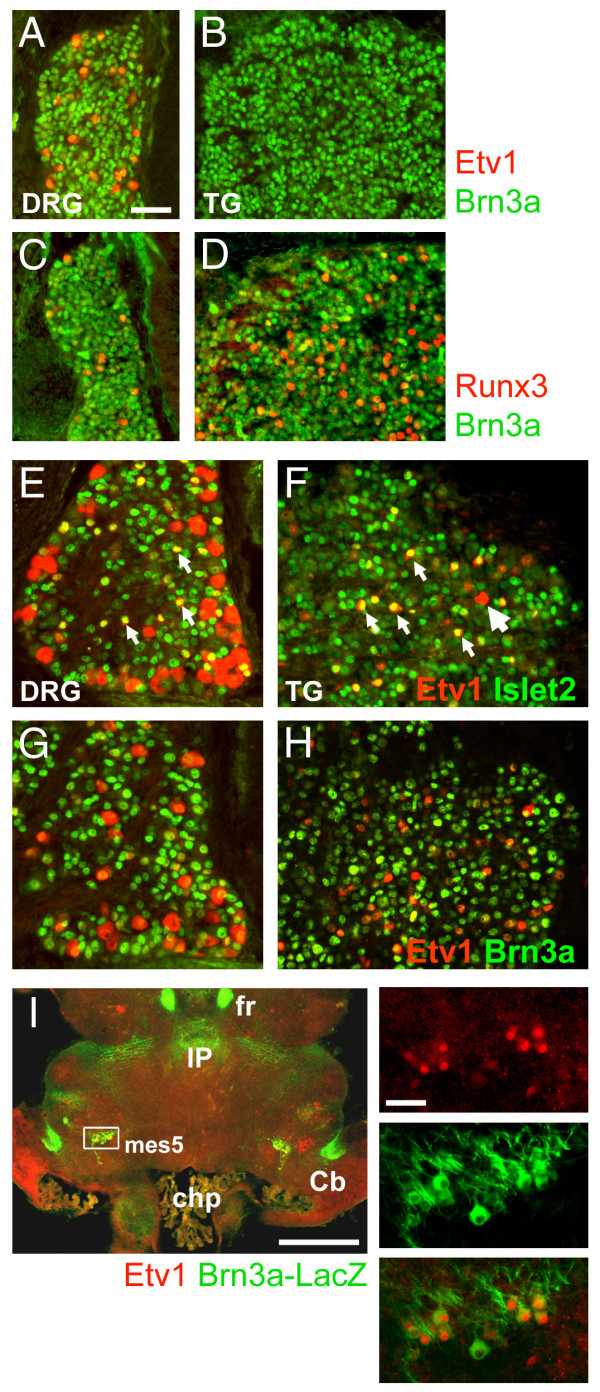
Selective expression of proprioceptor markers in the DRG and trigeminal system. The sensory ganglia of wild-type embryos were examined at E13.5 and E16.5 for the expression of transcription factors Brn3a, Etv1, Runx3 and Islet2. **(a-d) **At E13.5, Etv1 expression is restricted to the DRG, while Runx3 is expressed in both the DRG and TG. **(e-h) **At E16.5, Etv1 is expressed in both the DRG and the TG, but in the TG Etv1 positive neurons with large nuclei consistent with 1a proprioceptors are rare (large arrow). Instead, the majority of Etv1-positive neurons in the TG have nuclei of intermediate size and co-express Islet2; similar cells are also found in the DRG (small arrows). The 1a proprioceptors of the DRG co-express Brn3a, but at relatively low levels. **(i) **Etv1 expression in the mesV of an E18.5 embryo expressing a *tauLacZ *transgene integrated into the Brn3a locus [42], which is thus heterozygous for Brn3a, but phenotypically normal. Numerous neurons that co-express Etv1 and the Brn3a-LacZ marker are noted. The caudal location and large size of these neurons are consistent with proprioceptors innervating the muscles of mastication. Cb, cerebellum; chp, choroid plexus; fr, fasciculus retroflexus; IP, interpeduncular nucleus; mes5, mesencephalic trigeminal. Scale 50 μm (a-h), 400 μm (i), 50 μm (i, inset).

Aside from the Hox factors, the transcript with the greatest relative expression in the DRG was Etv1, an Ets-family transcription factor expressed in 1a muscle spindle afferents [13,21]. Immunofluorescence for Etv1 expression in the E13.5 DRG confirmed expression in sensory neurons of large and intermediate size, whilst expression was entirely absent in the TG at this stage (Figure 2a, b). In contrast, expression of Runx3, an earlier and more general marker of proprioceptive neurons [13,14], was noted in both the DRG and TG at E13.5 (Figure 2c, d).

By E16.5, Etv1 expression could be detected in some neurons of the TG, as well as the DRG. In the DRG, one population of Etv1-expressing neurons displayed large soma and nuclei consistent with 1a proprioceptors. However, in the TG nearly all of the Etv1-expressing neurons were of intermediate size, and large Etv1-positive neurons consistent with 1a proprioceptors were rare. In both the DRG and the TG, this intermediate-sized and later-developing population of Etv1-expressing sensory neurons could be further distinguished from the 1a proprioceptors by the co-expression of Islet2 (Figure 2e, f).

One distinguishing feature of the TG is that a subset of neurons that are functionally part of the trigeminal system, the mesV, is located within the CNS. Examination of the mesV in late gestation revealed large sensory neurons positive for Etv1. These neurons co-expressed a *tauLacZ *transgene integrated into the Brn3a locus, and Brn3a is a known marker of the mesencephalic trigeminal [6]. The position of these neurons in the caudal midbrain is consistent with the role of some of these neurons as muscle spindle afferents for the muscles of mastication [22]. Thus, the exclusive expression of Etv1 in the DRG but not the TG at E13.5 appears to result from a population of Etv1^+^, Islet2^- ^proprioceptive neurons that are present in the DRG but are largely restricted to the mesV at the cranial level.

### Brn3a regulates multiple downstream targets in the DRG

**Figure 3:**
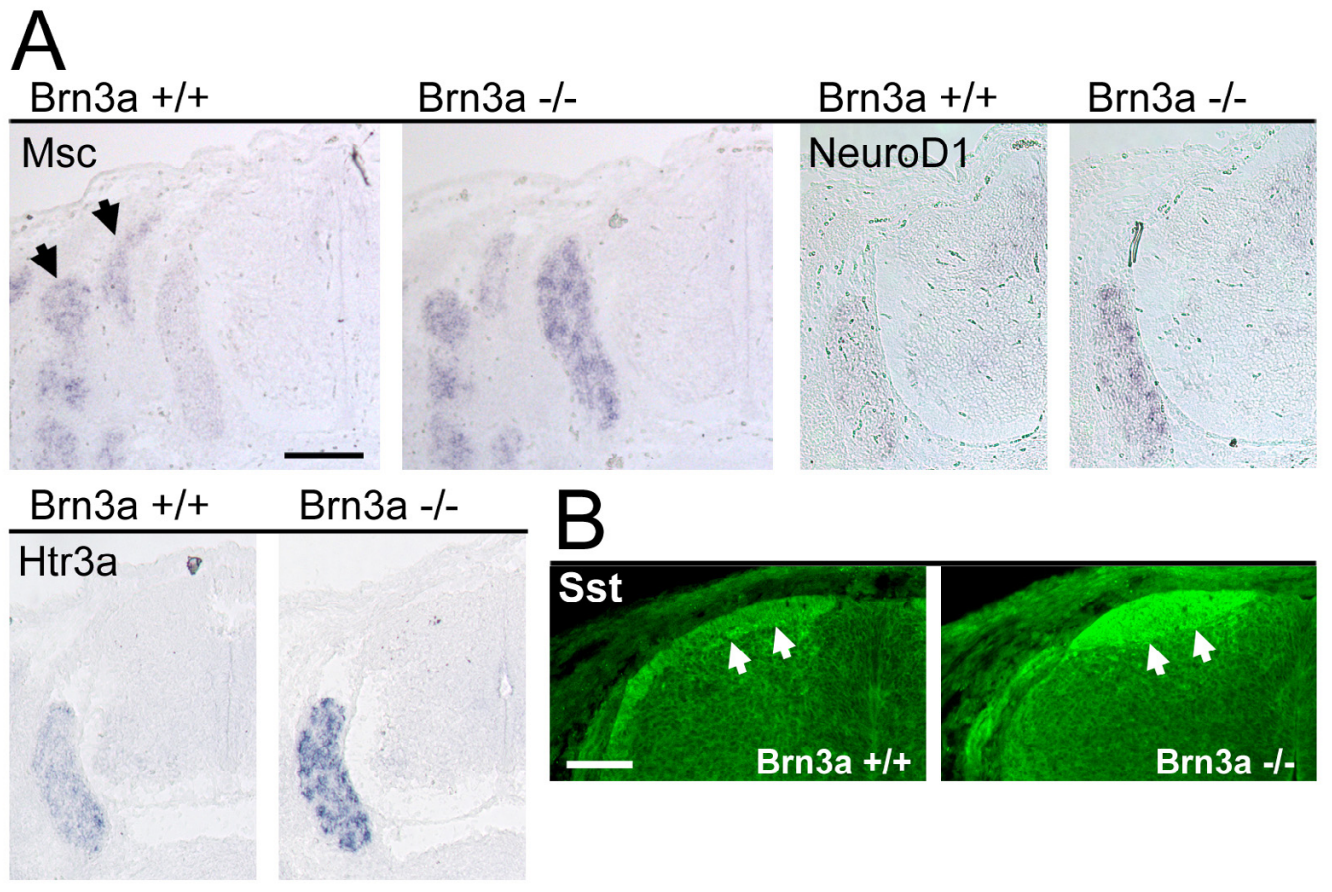
Target genes with increased expression in the DRG of Brn3a null mice. **(a) ***In situ *hybridization confirms increased expression of Msc (musculin), NeuroD1, and Htr3a (serotonin receptor 3A) transcripts in the DRG of Brn3a wild-type and knockout embryos. Note that the expression of Msc in the surrounding musculature (arrows) is unchanged. **(b) **Immunofluorescence for the somatostatin-14 peptide shows increased expression concentrated in the dorsal root entry zone (arrows). The distribution of somatostatin-14 also reveals an abnormal accumulation of axons in the superficial dorsal horn in the Brn3a null mutant. All views show lower cervical (brachial) level cross-sections of E13.5 embryos. Scale: 200 μm (a), 50 μm (b).

**Figure 4:**
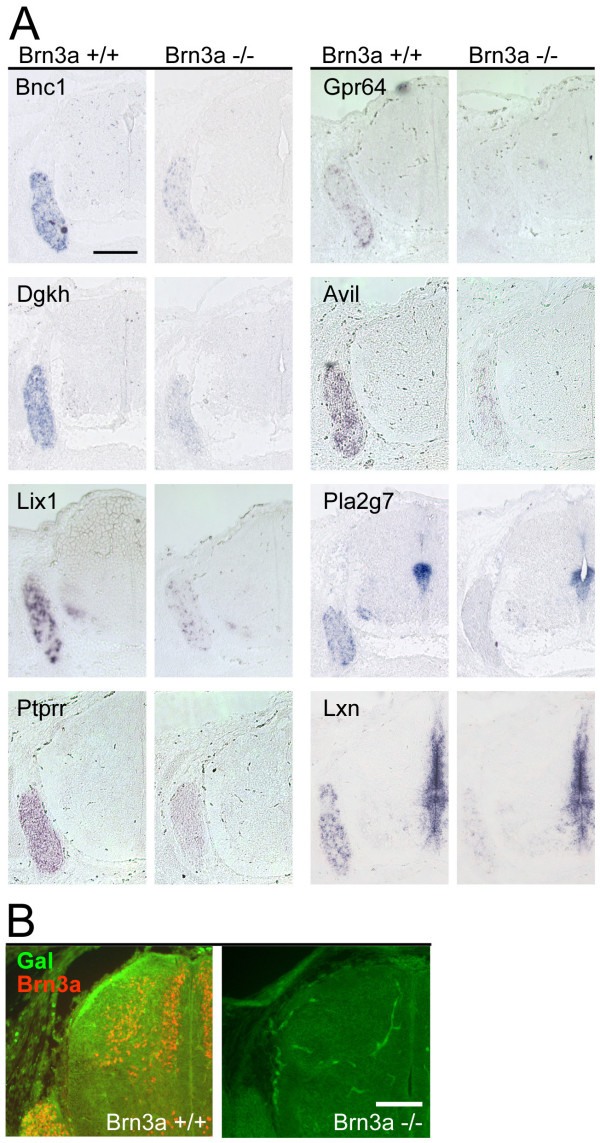
Target genes with decreased expression in the DRG of Brn3a null mice. **(a) ***In situ *hybridization confirms decreased expression of multiple Brn3a downstream targets. **(b) **Immunofluorescence for the galanin neuropeptide shows marked reduction in the dorsal horn and dorsal root entry zone of Brn3a null DRG. All views show lower cervical (brachial) level cross sections of E13.5 embryos. Scale: 200 μm (a), 100 μm (b).

To understand the developmental programs regulated by Brn3a at spinal versus cranial levels, we next analyzed global gene expression in E13.5 DRG from Brn3a mutant embryos and wild-type controls. Tables 2 and 3 summarize the transcripts most increased and decreased at E13.5 in the DRG of Brn3a knockout mice, and a more complete list appears in additional file 2. *In situ *hybridization and immunofluorescence were used to verify altered expression of several of the target genes (Figures 3 and 4), with an emphasis on newly identified Brn3a targets and genes for which expression has not been previously described in the sensory ganglia.

**Table 2:** Increased transcripts in the DRG of Brn3a knockout mouse

Transcript	Symbol	Class	WT	HT	KO	KO/WT
Chordin-like 1	Chrdl1	Dev	5	2	134	27.4
Musculin (MyoR)	Msc	TX	211	675	1,260	6.0
Insulinoma-associated 1	Insm1^2^	TX	153	301	827	5.4
GABA transporter 1 (Gabt1)	Slc6a1	NT	74	93	397	5.3
Microfibrillar-associated 4	Mfap4	Unk	112	370	559	5.0
Secretogranin II	Scg2	SY	206	253	966	4.7
C-fos induced growth factor (VEGF-D)	Figf	Dev	76	102	319	4.2
Guanylate cyclase 1, alpha 3	Gucy1a3	ST	50	82	205	4.1
Neurogenic differentiation 6 (Math2, Nex)	Neurod6	TX	83	200	344	4.1
Somatostatin	Sst	NT	215	382	830	3.9
Neurexin III	Nrxn3^4^	SY	367	961	1,412	3.8
Bruno-like 4	Brunol4^3^	Other	154	274	588	3.8
Cell adhesion molecule with homology to L1CAM	Chl1	AX	128	127	466	3.6
Junctophilin 1	Jph1	Other	74	119	262	3.5
Glutamate receptor, ionotropic, AMPA4	Gria4^3^	NT	46	80	161	3.5
Glutamate receptor, ionotropic, AMPA3	Gria3	NT	40	66	138	3.5
Zinc finger homeobox 1b	Zfhx1b^2^	TX	81	127	270	3.3
Nel-like 2	Nell2	AX	383	331	1,215	3.2
Follistatin-like 5	Fstl5^2^	Dev	281	389	885	3.2
Disabled homolog 1	Dab1^2^	AX	90	42	272	3.0
Mannan-binding lectin serine protease 1	Masp1	Other	95	101	282	3.0
**Selected**						
Semaphorin 3C	Sema3c^2^	AX	589	723	1,732	2.9
Cholecystokinin A receptor	Cckar	NT	95	161	269	2.8
Serotonin receptor 3A	Htr3a	NT	778	1,160	2,090	2.7
Homeobox A5	Hoxa5	TX	628	896	1,493	2.4
Neurogenic differentiation 1	Neurod1^2^	TX	1,048	1,468	2,440	2.3
p21-activated kinase 3	Pak3^2^	SY	246	270	568	2.3
Semaphorin 3D	Sema3d	AX	318	365	725	2.3
Protocadherin 17	Pcdh17^2^	AX	331	340	746	2.3
Spondin 1, (f-spondin)	Spon1	AX	629	778	1,413	2.2
K^+ ^voltage-gated channel, Isk family	Kcne1l	NT	613	813	1,328	2.2
Deleted in colorectal carcinoma	Dcc	AX	93	101	200	2.2
Netrin G1	Ntng1	AX	388	556	812	2.1
Neuropilin 2	Nrp2	AX	509	485	1,063	2.1
Cadherin 22	Cdh22	AX	199	226	414	2.1
Synapsin II	Syn2^4^	SY	643	688	1,324	2.1
Glutamate receptor, ionotropic, kainate 1	Grik1	NT	221	241	455	2.1
Protocadherin 8	Pcdh8^2^	AX	278	398	569	2.0
GDNF receptor alpha 2	Gfra2	Dev	363	436	735	2.0
Nescient helix loop helix 2 (Nscl2)	Nhlh2	TX	1,222	1,996	2,446	2.0

Data for one of two independent experiments are shown. All listed transcripts showed increased expression (change *p *< 0.005) in two independent replicates. Data are complete for all replicated changes above three-fold, and selected data appear for transcripts exhibiting two- to three-fold change. A full data set appears in Additional file 1. Superscript numerals represent the number of probe sets concordant for changed expression for a given gene. AX, neurogenesis/axon growth and guidance; Dev, development; KO, knockout; NT, neurotransmission; ST, signal transduction; SY, synapse formation and function; TX, transcription; Unk, unknown; WT, wild type.

**Table 3:** Transcripts decreased in the DRG of Brn3a null mice

Transcript	Symbol	Class	WT	HT	KO	WT/KO
Limb expression 1	Lix1	Unk	984	923	46	21.4
Vascular endothelial growth factor C	Vegfc	Dev	55	54	3	16.1
Neural cell adhesion molecule 2	Ncam2^2^	AX	280	212	22	12.9
Runt related 3	Runx3	TX	251	421	22	11.3
Brn3b	Pou4f2	TX	613	772	61	10.0
**Brn3a**	Pou4f1^2^	TX	1,107	1,119	115	9.6
Phospholipase A2, group VII	Pla2g7	ST	618	438	77	8.1
K+ channel, shaker-related, member 1	Kcna1^3^	NT	438	523	59	7.4
Advillin	Avil	AX	2,494	1,735	364	6.9
Galanin	Gal	NT	5,388	4,414	824	6.5
Basonuclin 1	Bnc1	TX	875	740	165	5.3
Insulin-like growth factor 1	Igf1^3^	Dev	820	609	160	5.1
G protein-coupled receptor 64	Gpr64^2^	NT	371	298	72	5.1
Adenylate cyclase activating polypeptide 1, PACAP	Adcyap1	NT	283	198	57	5.0
Regulator of G-protein signaling 10	Rgs10	NT	1,793	1,596	362	5.0
Parvalbumin	Pvalb	ST	403	223	95	4.3
K+ channel, shaker-related, beta member 2	Kcnab2	NT	952	684	238	4.0
Diacylglycerol kinase, eta	Dgkh^2^	ST	1,348	1,230	353	3.8
G protein-coupled receptor 73	Gpr73	NT	336	157	90	3.7
Serine proteinase inhibitor, clade A, member 3G; Spi2A	Serpina3g	Other	426	346	116	3.7
Copine IV	Cpne4	Unk	767	636	216	3.6
PQ loop repeat containing 1	Pqlc1	Unk	911	596	259	3.5
Reticulon 4 receptor-like 2; nogo receptor-like 3	Rtn4rl2	AX	789	694	226	3.5
Spermatogenesis associated glutamate-rich protein 1	Speer1-ps1	Unk	349	307	106	3.3
Pappalysin 2	Pappa2	Other	694	794	225	3.1
Brn3c	Pou4f3	TX	394	361	130	3.0
Protein tyrosine phosphatase, receptor type, J	Ptprj	ST	853	918	284	3.0
Docking protein 4	Dok4	ST	2,014	1655	679	3.0
**Selected**						
Eph receptor A7	Epha7	AX	206	202	77	2.7
Latexin	Lxn	NT?	2,948	2,564	1,105	2.7
DRG11	Prrxl1	TX	1,514	1,339	569	2.7
Protein tyrosine phosphatase, non-receptor type 3	Ptpn3	ST	1,638	1,881	623	2.6
GABA-A receptor, subunit alpha 2	Gabra2	NT	62	90	24	2.6
Na+ channel, type VII, alpha	Scn7a	NT	1,199	1,386	506	2.4
Protein tyrosine phosphatase, receptor type, R	Ptprr	ST	3,376	3,207	1,467	2.3
Rap1, GTPase-activating protein 1	Rap1ga1	ST	1,721	1,686	749	2.3
LIM domain binding 2	Ldb2	TX	794	492	349	2.3
Anthrax toxin receptor 2	Antxr2	Unk	1,355	1,095	608	2.2
Runt related txn factor 1	Runx1	TX	360	343	162	2.2
Homeobox D1	Hoxd1	TX	852	751	404	2.1
Inhibitor of DNA binding 1	Id1	TX	1,248	1,051	624	2.0

All listed transcripts showed decreased expression (change *p *> 0.995) in two independent replicates. Complete data appear for all replicated changes above three-fold, and selected data appear for transcripts exhibiting change in the two- to three-fold range. A full data set appears in Additional File 2. Note that Brn3a transcripts are still detected in the Brn3a null genotype, consistent with the detection of the 3'-end of residual non-coding transcripts. AX, neurogenesis/axon growth and guidance; Dev, development; KO, knockout; NT, neurotransmission; ST, signal transduction; SY, synapse formation and function; TX, transcription; Unk, unknown; WT, wild type.

Most of the Brn3a-regulated genes of known function have specific roles in neurotransmission, axonogenesis/synaptogenesis, signal transduction, regulation of developmental pathways, or transcription. Among mediators of neurotransmission, multiple glutamate receptors (Gria3, Gria4, Grik1), a GABA transporter (Slc6a1), a serotonin receptor (Htr3a) and the neuropeptide somatostatin are increased, while a GABA receptor (Gabra2) and neuropeptides with specific sensory roles (Galanin, Adcyap1/PACAP) are decreased. Also reduced is latexin, a secreted carboxypepidase inhibitor with a role in nociception [23].

Profound changes were also observed in genes that encode known or potential mediators of sensory neurogenesis, and axon growth or guidance. Increased transcripts in this class included the cell adhesion molecule Chl1, which has a known role in the growth of cortical dendrites [24], and Nel-like 2, an epidermal growth factor (EGF)-repeat containing factor with a previously demonstrated role in sensory development [25]. Increased expression was also noted for several molecules with known or likely roles in axon guidance, including Disabled1, Sema3c, Sema3d, f-spondin, Cadherin22, Protocadherin 8, Protocadherin 17, Dcc, and NetrinG1. Decreased expression was noted for advillin, an actin binding protein that is a known mediator of sensory neurite outgrowth [26], as well as Ncam2 and Eph receptor A7. These changes suggest that the profound defects observed in the sensory axons of Brn3a null mice [17] result from the derangement of a coordinated program of expression of mediators of axon growth and guidance, rather than from a single molecular lesion.

Loss of Brn3a also resulted in significant changes in the expression of other transcription factors. Notably, deletion of Brn3a effectively resulted in a triple-knockout of the Pou4 homeodomain class, because Pou4f2 (Brn3b) expression is nearly eliminated, and Pou4f3 (Brn3c) expression is greatly reduced in Brn3a knockout ganglia. Marked decreases were also observed in specific markers of sensory neuron subclasses, including Prrxl1/DRG11, Runx1 and Runx3. In contrast, specific bHLH genes, including those encoding NeuroD1, NeuroD6, Msc, and Nhlh2, were increased, and, perhaps consistent with this, the inhibitory bHLH factor Id1, which can act to oppose the action of neurogenic bHLH genes, was decreased. The significance of some of these changes in the hierarchy of gene regulation in developing sensory neurons is discussed below.

**Figure 5:**
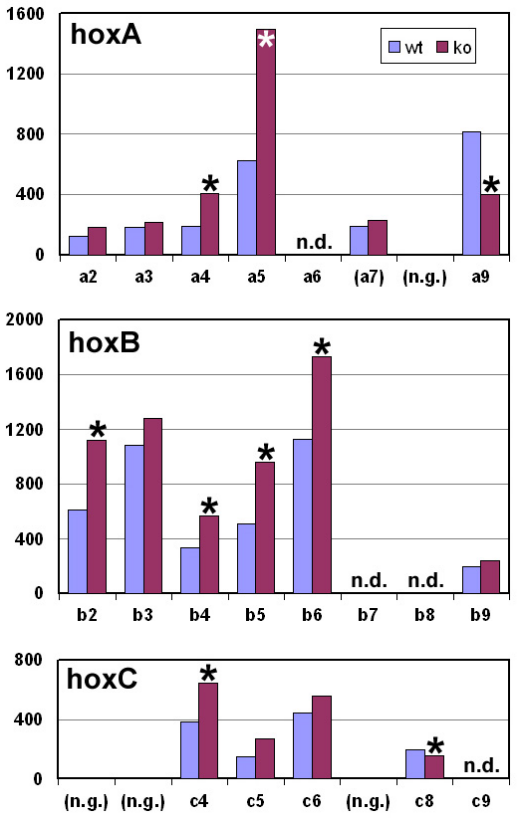
Brn3a regulation of *Hox *gene expression in the DRG. *Hox *gene expression data were derived from 430A+B array data for E13.5 Brn3a wild-type (wt) and knockout (ko) DRG. Statistically significant increases (*p *< 0.005) in the knockout were observed for 7 anterior Hox transcripts, and significant decreases were observed (*p *> 0.995) for two posterior Hox transcripts (asterisks). Similar changes reached statistical significance for 5 anterior *Hox *genes and one posterior *Hox *gene in a replicate assay. HoxA7 (parentheses) did not reach statistical significance for detection, indicating high background signal in this probe set. Legend: n.g., no gene at the corresponding position in the cluster; n.d., not determined.

Loss of Brn3a expression resulted in increased expression of essentially all of the anterior *HoxA*, *HoxB *and *HoxC *genes, and the changes reached statistical significance (*p *< 0.005 or *p *> 0.995, Materials and Methods) for seven genes in this group (Figure 5). The posterior *Hox *genes were expressed at much lower levels than the anterior factors, as expected for brachial-level DRG, and exhibited either unchanged or decreased expression in the knockout. Members of the HoxD cluster were not significantly expressed in the DRG (data not shown), with the exception of HoxD1, which, unlike the other anterior *Hox *gene products, exhibited decreased expression in Brn3a null DRG, and was also the only *Hox *gene product expressed at significant levels in the TG (see additional file 3) [20].

In prior studies, we have shown that Brn3a regulates its own expression via direct negative feedback, and that, in heterozygous Brn3a knockout mice, partial relief of this negative autoregulation results in increased expression of the intact allele, resulting in nearly complete gene dosage compensation. To determine whether gene dosage compensation is also present at spinal levels, we examined the levels of all increased and decreased transcripts in Brn3a^+/+^, Brn3a^+/- ^and Brn3a^-/- ^ganglia (see additional files 2 and 4). In each case, the mean effect of the loss of one *Brn3a *allele on target gene expression was 16% to 19% of the effect of the loss of both alleles, compared to the 50% change that would be expected without any compensatory mechanism, indicating significant but incomplete compensation for gene dosage.

### A subset of Brn3a targets are unique to the cranial sensory ganglia

Comparison of the regulatory targets of Brn3a in the developing DRG with our prior analysis of the TG [20] suggests that there are many downstream genes in common between these ganglia. However, results from the more advanced microarray used in the present study (Affymetrix 430) cannot be compared directly to the prior TG data derived from an older array (Affymetrix U74v2). To make such a comparison, we performed an additional analysis of these E13.5 DRG samples with the U74v2 array. We also updated the interpretation of the results for the TG U74v2 dataset using annotations from Build 34 of the mouse genome. This analysis confirmed that the DRG and TG of E13.5 Brn3a knockout mice have many conserved changes in gene expression (see additional file 3). Transcripts increased in both the DRG and TG of Brn3a knockout mice include the bHLH transcription factors musculin and NeuroD1, the neurotransmitters/receptors somatostatin, CCK-receptor A, 5HT-receptor 3A, and GABA-transporter 1, and the growth factor FIGF/VEGF-D. Conserved decreases include the transcription factors Hmx1, Runx1 and basonuclin, the peptides galanin and PACAP, as well as advillin and latexin.

**Figure 6:**
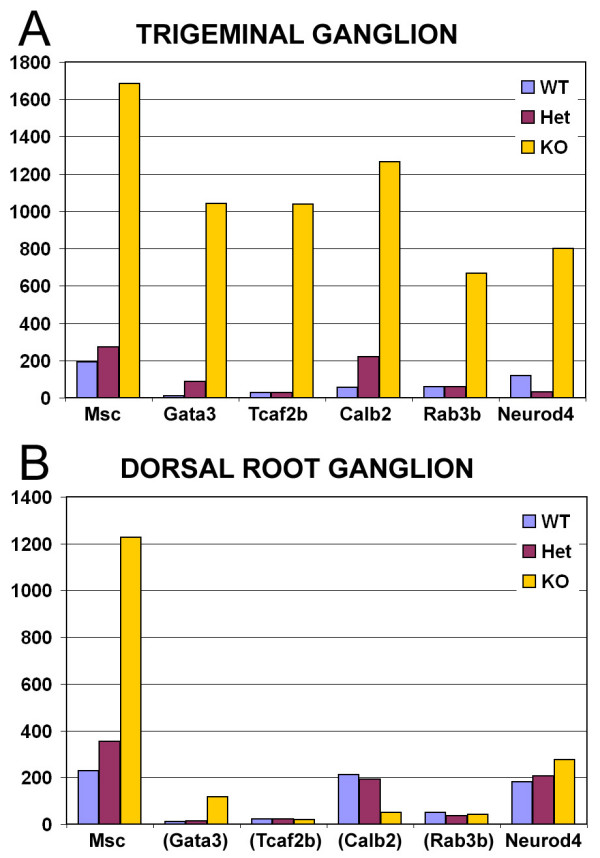
Trigeminal-specific targets of Brn3a regulation. **(a) **TG data from a previously published data set [20]. **(b) **DRG data from the present study. Brn3a wild-type (WT), heterozygote (Het) and knockout (KO) E13.5 DRG were analyzed for global gene expression using U74Av2+U74v2B arrays to allow direct comparison with the prior analysis of the embryonic TG (Additional file 3). Msc, shown for comparison, exhibits markedly increased expression in the DRG and TG of Brn3a knockout mice, while Gata3, Tcfab2b, Calb2, Rab3b and Neurod4 are increased only in the DRG. Changes in Msc, Gata3, Tcfap2b, Calb2 and NeuroD4 have been previously verified in the TG of Brn3a knockout mice by *in situ *hybridization or immunofluorescence [20]. Rab3b is newly identified as a Brn3a target in the TG due to progress in the annotation of the mouse genome. Gene abbreviations in parentheses indicate expression below the statistically significant threshold of detection (absent call) in all three genotypes.

Although the majority of the downstream targets of Brn3a were conserved in the DRG and TG, a subset of genes was uniquely regulated at each axial level. The most profound differences were observed for a set of genes markedly increased in the TG of Brn3a knockout embryos, and unchanged in the DRG (Figure 6). These differentially regulated genes include those encoding the transcription factors Tcfap2b (Ap2β), NeuroD4 (Math3) and Gata3, the calcium binding protein Calb2 (calretinin) and the small GTP-binding protein Rab3b. These results led us to consider possible mechanisms for cell-specific target gene regulation by Brn3a in these two closely related neural tissues.

The cell-type specific effects of transcription factors are generally attributed to distinct complements of interacting partners, or to the expression of related factors that provide redundancy in some cell types but not in others. However, with the exception of the Hox factors, the very similar gene expression profiles of the DRG and TG do not suggest many candidates for distinct Brn3a partners in these neurons. In addition, the loss of Brn3a effectively eliminates expression of all Pou4 class factors in both the DRG and TG, making selective redundancy unlikely.

**Figure 7:**
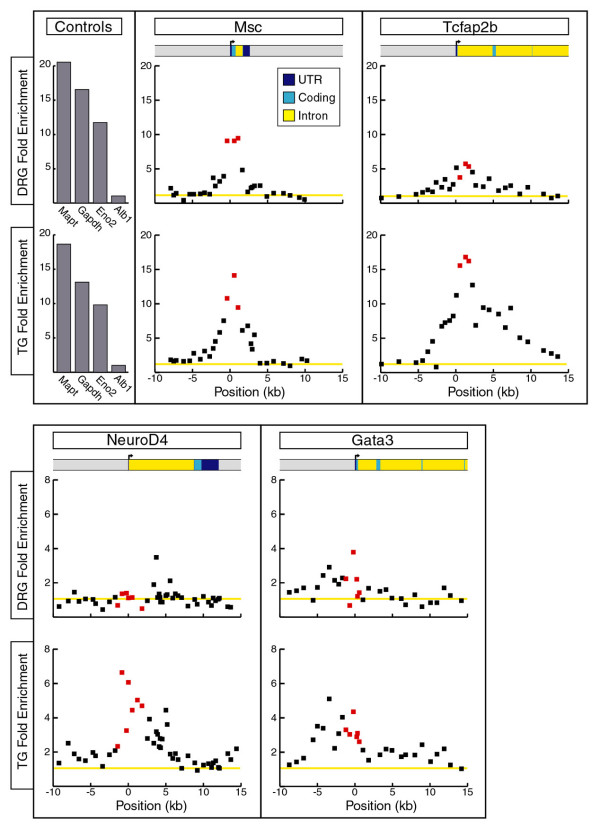
Acetyl-histone H3 profiling of Brn3a target gene loci. Histone H3 acetylation was assayed using ChIP and tiled PCR primer pairs from -10 kb to 15 kb of the *Msc*, *Tcfap2b*, *NeuroD4 *and *Gata3 *gene loci. Positive controls for these assays included primer pairs located in the promoter regions of the *Mapt *(tau), *Gapdh*, and *Eno2 *loci, which are highly expressed in the DRG and TG and are unchanged in Brn3a null mice. The silent *alb1 *locus was used as a negative control and to set the baseline of one-fold enrichment (yellow line). H3 acetylation of these loci in chromatin samples from the E13.5 DRG and TG were compared by *t*-tests using fold enrichment values for primer pairs flanking the transcription start site of each locus (points shown in red). H3 acetylation was not significantly different at the msc locus (*p *= 0.25), while *Tcfap2b *(*p *= 0.0003) and *NeuroD4 *(*p *= 0.0005) showed significantly greater acetylation in the TG, and *Gata3 *(*p *= 0.09) showed a trend toward greater acetylation in the TG. UTR, untranslated region.

One approach to understanding the cell-specific effects of the loss of Brn3a expression is suggested by recent work in which we have demonstrated a correlation between Brn3a binding to its target sites *in vivo *and H3-acetylation in the vicinity of the potential binding sites [[27] in press]. Given these results, one potential mechanism for the differential regulation of target genes by Brn3a might be distinct modifications of chromatin at the target gene loci in the DRG and TG, which could modulate the effects of Brn3a or downstream regulatory factors. To examine this question for the differentially regulated loci Tcfap2b, NeuroD4 and Gata3, we used chromatin immunoprecipitation (ChIP) to profile histone H3 acetylation in wild-type E13.5 DRG and TG (Figure 7). We also examined the Msc locus as an example of a gene that shows similarly increased expression in the DRG and TG of Brn3a knockout mice. ChIP assays of the promoter regions of three constitutively expressed genes, *Gapdh*, *Mapt *(tau), and *Eno2 *(neuron specific enolase), were used as positive controls, and the results were normalized to negative control assays from the promoter region of the *Alb1 *(albumin) gene, which is not expressed in the nervous system. Because the genomic sequences that regulate these genes in the sensory ganglia have not been defined, we surveyed histone acetylation across these loci using multiple oligonucleotide pairs spaced at 500 to 1,000 base-pair (bp) intervals from approximately -10 kb to +15 kb relative to the start of transcription, using quantitative real-time PCR ('locus-ChIP') ["locus-ChIP", [27] in press].

ChIP profiling of the Msc locus revealed similar levels of H3-acetylation in the DRG and TG. However, ChIP assays of the *Tcfap2b *and *NeuroD4 *loci revealed significantly greater H3-acetylation in the TG relative to the DRG (*p *= 0.0003 and *p *= 0.0005, respectively), while the Gata3 locus exhibited a trend (*p *= 0.09) toward greater acetylation in the TG. As expected, the positive control promoters were highly acetylated, and the negative control, *Alb1*, was deacetylated in both the DRG and TG. Thus, the state of H3-acetylation in wild-type ganglia appears to be correlated both with basal expression and also with a state of latent potential expression that can be induced in Brn3a null ganglia.

## Discussion

Numerous transcription factors have been shown to play key roles in the differentiation of brainstem, spinal, and spinal sensory neurons, yet, for the most part, the programs of gene expression regulated by these factors remain unknown. A layer of complexity is added to the analysis of the downstream targets of these factors by the fact that their expression patterns are often very complex, and may include diverse classes of neurons and non-neuronal cell types with no obvious common characteristics. Brn3a, for example, is expressed in a majority of differentiating peripheral sensory neurons, and also in specific neurons of the spinal cord, olivo-cerebellar system, midbrain, diencephalon and retina. The LIM-domain transcription factor Islet1 is co-expressed with Brn3a in the sensory system [10], but in the CNS it is expressed primarily in motor neurons [28,29], and also has important roles in the development of the heart [30] and pancreatic islet cells [31]. A fundamental unanswered question is whether these factors, and many others with expression patterns of similar complexity, regulate the same downstream targets in different neuronal types, and in neurons versus non-neuronal cells.

To better understand the molecular pathways of sensory development, we initiated the present study by examining global gene expression in the brachial-level DRG and TG at E13.5. At this stage, when essentially all sensory neurons have exited the cell cycle and markers of sensory subtypes are beginning to be expressed, the gene expression patterns of the DRG and TG are quite similar. Some transcripts exhibit quantitative differences between the ganglia, which may represent relative differences in cellular composition or the timetable of development, but very few transcripts are uniquely expressed in either the DRG or TG. Most prominent among the few transcripts restricted to a particular axial level are those encoded by the *Hox *genes and Etv1, which at this stage are expressed only in the DRG. The restriction of the Hox transcripts (with the exception of HoxD1) to spinal levels is expected based on the well characterized axial expression pattern of these genes. Etv1 expression is restricted to the DRG at E13.5 because the first neurons to express this marker, the large proprioceptive neurons innervating muscle spindles, develop within the DRG at spinal levels but at cranial levels are largely sequestered in the mesV. Etv1-expressing neurons that develop in the TG by E16.5 are distinguished by smaller size and the co-expression of Islet2, and appear to represent a distinct subset of sensory neurons.

Global analysis of gene expression in Brn3a knockout and wild-type DRG demonstrates that Brn3a regulates, directly or indirectly, an extensive program of gene expression. Advances in microarray technology and in the annotation of the mouse genome have allowed us to significantly expand the number of identified Brn3a targets from a prior study of the TG [20]. A large number of the downstream targets of Brn3a in the DRG have known roles in neurogenesis or neural function, and include components of axons and synapses, neurotransmitters and their receptors, mediators of intracellular signaling, and transcription factors. Because the regulatory sequences of many of these genes have not been described, in many cases it is not possible to distinguish direct regulatory targets from secondary or compensatory effects. However, in recent studies we have used locus-ChIP to demonstrate that Brn3a is a direct repressor of NeuroD1 and NeuroD4 in the embryonic TG [[27] in press], as well as a negative modulator of its own expression [32,33].

Most Brn3a target genes are conserved between sensory neurons at cranial and spinal levels. However, a subset of genes with the most increased expression in the TG do not change expression in the Brn3a knockout DRG, and are generally undetectable in the E13.5 DRG of animals of any genotype. These differentially regulated transcripts include the transcription factors Tcfap2b, Gata3 and NeuroD4, the calcium binding protein calbindin2 (calretinin), and the small GTP-binding protein Rab3b, implicated in synaptic vesicle release [34]. Because there are normally few transcripts that distinguish the TG from the DRG at this stage of development, the gene expression patterns of the DRG and TG are significantly more different in Brn3a knockout ganglia than in the wild type. This suggests that one important role for Brn3a is to suppress potential differences in gene expression between the spinal and cranial ganglia, mediating a process of 'convergent development' in which functionally similar populations of neurons are generated from different embryological sources.

The state of histone H3 acetylation of Brn3a target gene loci offers some insight into the underlying mechanism of the differential regulation of these targets at spinal and cranial levels. Normally, H3 acetylation is associated with the regulatory sequences in the promoters of actively transcribed genes. Consistent with this, we have previously shown that the promoter region of *Pou4f1 *itself is highly acetylated in the TG, and the promoters of genes that are silent in the sensory ganglia regardless of Brn3a genotype are deacetylated [[27] in press]. In wild-type DRG and TG, the differentially regulated genes *NeuroD4 *and *Tcfap2b *are nearly silent, yet these gene loci can be distinguished in trigeminal neurons by increased H3 acetylation over a region encompassing the transcription start site. The *Msc *gene is also normally silent, but it exhibits increased expression at both cranial and spinal levels in Brn3a null mice, and is accordingly H3 acetylated in both the DRG and TG. Thus, in the case of these genes with increased expression in the Brn3a knockout, H3 acetylation appears to reveal a latent state of potential expression, which is normally repressed by Brn3a or its downstream effectors, and de-repressed in Brn3a knockout ganglia.

The failure of DRG neurons to increase *NeuroD4 *and *Tcfap2b *expression in Brn3a knockout ganglia suggests that a redundant mechanism of repression exists for these genes at spinal but not cranial levels. The action of such a repressor may be reflected in the low levels of histone acetylation at these loci in the DRG. Because the most prominent distinguishing feature of sensory gene expression at spinal levels is the expression of multiple *Hox *genes, these factors are candidate mediators of this selective repression of *NeuroD4 *and *Tcfap2b *in the DRG. Transcriptional repressor functions have been described for the *Hox *genes [35], and there is evidence that Hox proteins can directly block the activity of the widely expressed histone acetyltransferase CBP-p300 [36]. *Hox *gene repression of a subset of Brn3a target gene loci could be an active process at E13.5, or *Hox *gene expression at earlier developmental stages could produce persisting modifications of chromatin, reflected in the deacetylation of these loci observed here, resulting in reduced transcription even when direct repression by *Hox *genes is no longer active.

## Materials and methods

### Matings, embryos, and RNA isolation for array analysis

To generate tissue for microarray analysis, timed matings of Brn3a heterozygote animals were performed, and the embryos were harvested at E13.5. Only embryos corresponding to E13.5 ± 0.5 days based on the staging system of Theiler [37] were used for microarray analysis. TG were removed by blunt dissection and carefully freed of adherent non-neural tissue with fine forceps. DRG were isolated by stripping of the spinal cord with its adherent ganglia, followed by dissection of the ganglia with fine forceps. DRG were harvested from brachial region only, including the C5-T1 levels. Dissected ganglia were placed in RNAse inhibitor solution (RNAlater, Ambion, Austin TX), and RNA was prepared using the RNeasy system (Qiagen, Valencia, CA). Embryos were genotyped for Brn3a alleles as previously described [17] from a sample of tail or hind-limb tissue harvested at the time of ganglion dissection. Genotyped TG or DRG from five embryos were sufficient to provide approximately 5 μg of total RNA for a single microarray analysis. The generation of cDNA, production of labeled cRNA, and hybridization to GeneChip arrays were all performed according to standard protocols provided by the manufacturer (Affymetrix, Santa Clara, CA).

### Analysis of expression array data

The principal microarray datasets presented here for the mouse DRG and TG were generated using the Affymetrix 430A and 430B microarrays. In addition, the same DRG samples were analyzed using Affymetrix U74A and U74B arrays for direct comparison with prior data sets for the TG [20]. The primary analysis of microarray data, including determination of the absence/presence of the assayed transcripts, transcript expression levels, and the probability of change in transcript expression between samples ('change *p*') was performed with Microarray Suite 5.0 (Affymetrix). Default Microarray Suite 5.0 parameters were used for increase (I) and decrease (D) calls. For the 430 array set these cutoff values were *p *< 0.005 and *p *> 0.995 for I and D, respectively, and for the U74 arrays, the values were *p *< 0.003 and *p *> 0.997. All array values were initially scaled to a mean value of 500 using global scaling. To permit more meaningful comparison of the expression levels of transcripts assayed by the 430A and 430B arrays, the initial expression values for the 430B array were rescaled based on the expression levels of 100 probe sets in common between the 430A and 430B arrays. Microarray probe sets were related to the corresponding mouse transcripts using the NetAffx database (Affymetrix), based on the NCBI Build 34 annotation of the mouse genome. The array data discussed in this publication have been deposited in NCBI's Gene Expression Omnibus (GEO) [38] and are accessible through GEO series accession number GSE5658.

Comparisons of gene expression between wild-type DRG and TG (Table 1) and between the DRG from Brn3a wild-type, heterozygous, and knockout mice (Tables 2 and 3) were performed in duplicate, using separate pools of isolated ganglia. To be included in the tables of changed transcripts for a given tissue or genotype, the following criteria were met: both replicates were called as 'present' in the condition of greater expression; both replicates exhibited an increased or 'I' call for the condition of higher expression; and the probe set identified an annotated and named transcript in NCBI Build 34 of the mouse genome.

Duplicate probe sets identifying the same transcript were eliminated, and the tables are annotated with the number of concordant probe sets for each transcript. Concordance was identified by the agreement of the present and increased calls for the duplicate probe set, and a minimum fold change of 2.0. In the case of multiple concordant probe sets, the probe set with the greatest fold change is listed in the tables. Results for the probe sets meeting the inclusion criteria were ranked by fold change, and the cutoff value for inclusion appears in the tables.

### *In situ *hybridization and immunofluorescence

Non-isotopic *in situ *hybridization was performed as previously described [38]. A list of probes used and their sources appears in additional file 5. Immunofluorescence for Brn3a was performed with rabbit polyclonal antisera as previously as previously described [39]. Other antisera used included rabbit antisera against Etv1/Er81 and Runx3 obtained from Dr Sylvia Arber [13,15], and guinea pig antisera against Islet2, obtained from Dr Sam Pfaff [28]. Immunofluorescence for other antigens was performed with commercially available antibodies, including rabbit anti-calretinin (Swant, Bellinzona, Switzerland), rabbit anti-galanin (Bachem, King of Prussia, PA), rabbit anti-somatostatin-14 (Peninsula Laboratories), and goat anti-β-galactosidase (Biogenesis (MorphoSys), Kingston, NH).

### Locus-wide chromatin immunoprecipitation

Locus-ChIP assays were performed as previously described [[27] in press]. In brief, embryos for ChIP assays were generated from timed matings of ICR mice. DRG and TG were dissected from E13.5 embryos and fixed in 4% paraformaldehyde for 30 minutes, then quenched with 150 mM glycine. The fixed tissue was washed with phosphate-buffered saline and stored at -80°C until analysis.

Selection of chromatin complexes from embryonic sensory ganglia was performed by a modification of a widely used procedure [40]. For each analysis, fixed ganglia from 30 embryos (60 TG or approximately 300 DRG) were pooled and suspended in lysis buffer containing 50 mM Tris-HCl, pH 8.1, with 10 mM EDTA and 1% SDS, 1 mM 4-(2-Aminoethyl)benzenesulfonylfluoride, HCl (AEBSF) and a proprietary protease inhibitor mix (1 × Complete Mini, Roche, Indianapolis, IN; used according to instructions). Chromatin was then fragmented to an average size of 500 bp by sonication, and insoluble cellular debris was removed by centrifugation. The supernatant containing fragmented chromatin was diluted in 15 mM Tris-HCl, pH 8.1, with 150 mM NaCl, 1 mM EDTA, 1% Triton-X-100, 0.01% SDS, and protease inhibitor mix. An unselected ('input') sample of 10% of the total homogenate was removed prior to antibody selection.

ChIP was performed using rabbit anti-acetylated histone H3 antibody (Upstate Biotechnology, Inc., Bellerica, MA, catalog no. 06–599), which recognizes histone H3 acetylated at lys9 and lys14. For each selection, 50 μg of anti-histone antibody was coupled to 250 μL of anti-rabbit IgG magnetic beads (Dynal M-280), in lysis buffer containing 50 mM Tris-HCl, pH 8.1, with 10 mM EDTA and 1% SDS. To reduce non-specific background, the chromatin sample was pre-cleared using the magnetic beads with the secondary antibody alone. The sample was then incubated overnight with secondary antibody-coupled beads to select the acetyl H3-containing chromatin complexes. The beads were then washed for 5 minutes at room temperature with each of the following solutions: 20 mM Tris-HCl, pH 8.1, with 150 mM NaCl, 2 mM EDTA, 0.1 % SDS, and 1% Triton-X-100; 20 mM Tris-HCl, pH 8.1, with 500 mM NaCl, 2 mM EDTA, 0.1% SDS, and 1% Triton-X-100; 10 mM Tris-HCl, pH 8.1, with 1 mM EDTA, 0.25 M LiCl, 1% NP-40, and 1% deoxycholate. Salt and detergent were removed by washing twice with 10 mM Tris-HCl, 1 mM EDTA, pH 8. DNA was extracted from the antibody-chromatin complexes and the input sample by heating in 0.1 M NaHCO_3 _with 200 mM NaCl and 1% SDS at 65°C for 4 hours with constant shaking. The input and selected samples were then digested with proteinase K, extracted with phenol/chloroform, and precipitated with ethanol.

### Real-time locus-wide PCR analysis

Chromatin fragments recovered from the immunoprecipitated and input samples were then assayed by real-time PCR using an ABI 7300 thermocycler and SYBR Green fluorimetric detection. To screen an entire gene locus, oligonucleotide pairs were designed at 500 to 1,000 bp intervals throughout the region, and selected and unselected samples were run in parallel in a 96-well plate format. The primer pairs used for locus-ChIP assays of the *NeuroD4*, *Msc*, *Tcfap2b *and *Gata3 *loci appear in additional file 5.

The enrichment of immunoprecipitated chromatin fragments was assayed by the cycle-threshold difference method [41]. For this method, real-time PCR signals are measured using the 'cycle threshold', or Ct parameter, which is the number of cycles required for the amplification product to reach an arbitrary level of fluorescence intensity (threshold), and is logarithmic to the initial abundance of the target sequence in the sample. For each PCR amplicon, a ΔCt value comparing the unselected (input) and antibody-selected DNA samples was then calculated by subtracting the Ct_selected _from the Ct_input _signal:

ΔCt = Ct_input _- Ct_selected_

Fold enrichment values for target sequences bound by the selecting antibodies, corresponding to the y-axis of the locus-ChIP plots, were calculated using the following equation:

E = 2^(ΔCt-ΔCtcontrol)^

Because product formation approximately doubles with each cycle in the linear range of amplification, a ΔCt of one cycle represents a two-fold difference in starting template. A significant advantage of this method is that, for each primer pair, a selected sample is compared directly to its unselected control, which differs only by the antibody selection process. Potentially confounding factors such as small differences in the PCR amplification efficiency of different primer pairs are eliminated in this comparison.

The ΔCt assays for each pool of selected material were normalized to an arbitrary baseline (one-fold enrichment) determined using two primer pairs in the promoter region of the *Alb1 *(albumin locus), which was chosen as a negative control because it is not transcribed in the nervous system. Fold enrichment values for the *Alb1 *locus were very similar to values for the unselected, intergenic regions of the target gene loci. Primer pairs in the promoter regions of *Mapt *(microtubule-associated protein tau) and *Eno2 *(neuron specific enolase), which exhibit tissue specific expression in the nervous system, and *Gapdh *(glyceraldehyde-3-phosphate dehydrogenase), which is expressed ubiquitously, were used as positive controls. Comparisons of histone H3 acetylation at a given locus in the DRG and TG were made using three- to six-fold enrichment values using oligonucleotide pairs in a contiguous genomic region near the transcription start site of the analyzed gene loci. The statistical significance (*p *values) of the difference in H3 acetylation was determined using two-sample, unequal variance *t*-tests.

## Competing interests

The author(s) declare that they have no competing interests.

## Supplementary Material

Additional file 1 Differential gene expression in trigeminal and dorsal root ganglia. In situ hybridization showing transcripts differentially expressed in the trigeminal and dorsal root ganglia. Click here for file

Additional file 2 Increased and Decreased transcripts in E13.5 DRG of Brn3a knockout mice. This shows a more complete version of the data set presented in Table 1. Click here for file

Additional file 3 Direct comparision of altered gene expression in DRG and TG of Brn3a knockout sensory ganglia. Gene expression in the DRG is analyzed using the U74v2 array set to allow direct comparision to a prior data set for the TG. Click here for file

Additional file 4 Dorsal root ganglia exhibit significant compensation for the loss of one Brn3a allele. Gene expression levels are compared for Brn3a wild-type, heterozygote, and knockout ganglia to demonstrate the extent to which gene dosage compensation reduces the heterozygote phenotype. Click here for file

Additional file 5 Summary of insitu hybridization probes andLocus-ChIP oligonucleotides. Summary of insitu hybridization probes andLocus-ChIP oligonucleotides. Click here for file
