# Supplementary material for: POU-domain factor Brn3a regulates both distinct and common programs of gene expression in the spinal and trigeminal sensory ganglia
Source: Neural Dev. 2007 Jan 19;2:3. doi: 10.1186/1749-8104-2-3 (PMC1796875; doi:10.1186/1749-8104-2-3)
Supplement: Additional file 1 — Differential gene expression in trigeminal and dorsal root ganglia. In situ hybridization showing transcripts differentially expressed in the trigeminal and dorsal root ganglia. [file 1749-8104-2-3-S1.doc]

**Additional file 1**

**Differential gene expression in trigeminal and dorsal root ganglia.**

Expression was assessed by in situ hybridization in E13.5 embryos. (A-D) Transcripts exhibiting greater expression in the DRG. (E-F) Transcripts exhibiting greater expression in the DRG, with a predominantly glial expression pattern. Arrows indicate the peripheral nerve roots of the DRG which are relatively enriched in glia. (G-M) Transcripts with greater expression in the TG. Lgals7 is expressed in the sensory ganglia (TG>DRG, K,L) and also in the whisker follicles (M). Dien, diencephalon; SC, spinal cord. Scale (A-L) 50μm, (M) 200μm.

**
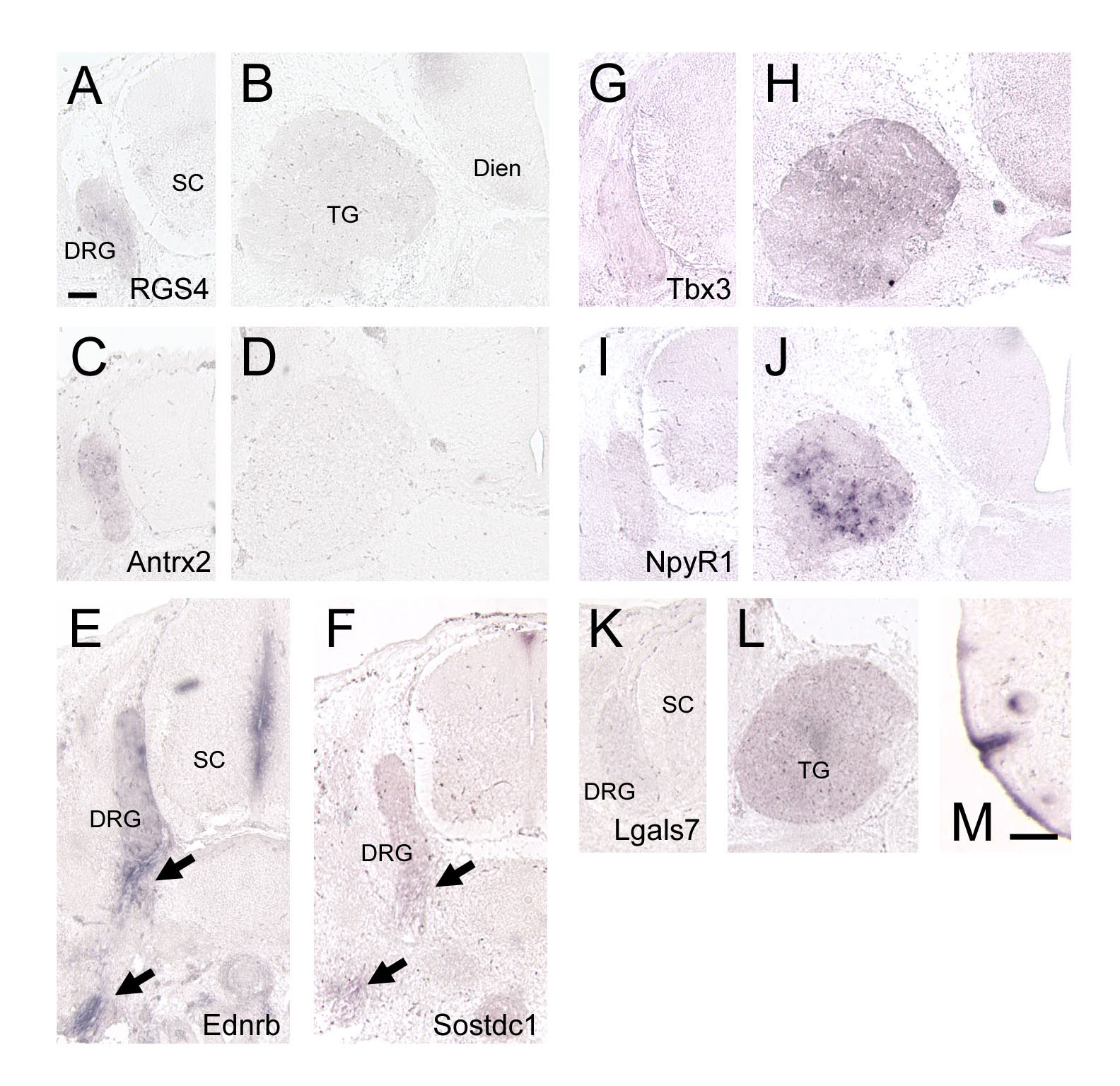
**
