# Supplementary material for: POU-domain factor Brn3a regulates both distinct and common programs of gene expression in the spinal and trigeminal sensory ganglia
Source: Neural Dev. 2007 Jan 19;2:3. doi: 10.1186/1749-8104-2-3 (PMC1796875; doi:10.1186/1749-8104-2-3)
Supplement: Additional file 2 — Increased and Decreased transcripts in E13.5 DRG of Brn3a knockout mice. This shows a more complete version of the data set presented in Table 1. [file 1749-8104-2-3-S2.doc]

**Additional file 2**

**2.1 Increased transcripts in E13.5 DRG of Brn3a knockout mice.** Superscript numerals indicate number of probe sets showing significant change for a specific transcript. (*) indicates transcripts previously reported to be changed in the trigeminal ganglia of E13.5 embryos.

|  |  |  | **Experiment** | | | | **Replicate** | | | |
| --- | --- | --- | --- | --- | --- | --- | --- | --- | --- | --- |
| **Gene Title** | **Symbol** | **Class** | **WT** | **HT** | **KO** | **KO/WT** | **WT** | **HT** | **KO** | **KO/WT** |
| Chordin-like 1 | Chrdl1 | Dev | 5 | 2 | 134 | 27.4 | 18 | 12 | 92 | 5.0 |
| Musculin; MyoR* | Msc | TX | 211 | 675 | 1260 | 6.0 | 303 | 469 | 994 | 3.3 |
| Insulinoma-associated 1 | Insm1^2^ | TX | 153 | 301 | 827 | 5.4 | 131 | 207 | 611 | 4.7 |
| GABA transporter 1, Gabt1 | Slc6a1 | NT | 74 | 93 | 397 | 5.3 | 58 | 125 | 284 | 4.9 |
| Microfibrillar-associated 4 | Mfap4 | Unk | 112 | 370 | 559 | 5.0 | 60 | 224 | 492 | 8.2 |
| Secretogranin II | Scg2 | SY | 206 | 253 | 966 | 4.7 | 218 | 235 | 628 | 2.9 |
| C-fos induced growth factor; VEGF-D* | Figf | Dev | 76 | 102 | 319 | 4.2 | 77 | 106 | 274 | 3.6 |
| Guanylate cyclase 1, alpha 3 | Gucy1a3 | ST | 50 | 82 | 205 | 4.1 | 38 | 41 | 166 | 4.4 |
| Neurogenic differentiation 6 (Math2, Nex) | Neurod6 | TX | 83 | 200 | 344 | 4.1 | 150 | 123 | 374 | 2.5 |
| Somatostatin* | Sst | NT | 215 | 382 | 830 | 3.9 | 295 | 394 | 851 | 2.9 |
| Neurexin III | Nrxn3^4^ | SY | 367 | 961 | 1412 | 3.8 | 862 | 708 | 1130 | 1.3 |
| Bruno-like 4* | Brunol4 (3) | Other | 154 | 274 | 588 | 3.8 | 113 | 202 | 420 | 3.7 |
| Cell adhesion molecule with homology to L1CAM | Chl1 | AX | 128 | 127 | 466 | 3.6 | 158 | 171 | 287 | 1.8 |
| Junctophilin 1 | Jph1 | Other | 74 | 119 | 262 | 3.5 | 99 | 95 | 204 | 2.1 |
| Glutamate receptor, ionotropic, AMPA4 | Gria4^3^ | NT | 46 | 80 | 161 | 3.5 | 83 | 76 | 173 | 2.1 |
| Glutamate receptor, ionotropic, AMPA3 | Gria3 | NT | 40 | 66 | 138 | 3.5 | 39 | 58 | 136 | 3.5 |
| zinc finger homeobox 1b | Zfhx1b^2^ | TX | 81 | 127 | 270 | 3.3 | 120 | 69 | 262 | 2.2 |
| Nel-like 2* | nell2 | AX | 383 | 331 | 1215 | 3.2 | 414 | 380 | 1144 | 2.8 |
| Follistatin-like 5 | Fstl5^2^ | Dev | 281 | 389 | 885 | 3.2 | 326 | 468 | 675 | 2.1 |
| Disabled homolog 1 | Dab1^2^ | AX | 90 | 42 | 272 | 3.0 | 33 | 108 | 278 | 8.4 |
| Mannan-binding lectin serine protease 1 | Masp1 | Other | 95 | 101 | 282 | 3.0 | 117 | 144 | 256 | 2.2 |
| Semaphorin 3C | Sema3c^2^ | AX | 589 | 723 | 1732 | 2.9 | 788 | 583 | 1343 | 1.7 |
| Solute carrier family 35, member F1 | Slc35f1^2^ | Unk | 585 | 881 | 1694 | 2.9 | 996 | 669 | 1931 | 1.9 |
| Cholecystokinin A receptor | Cckar | NT | 95 | 161 | 269 | 2.8 | 95 | 135 | 203 | 2.1 |
| serotonin receptor 3A* | Htr3a | NT | 778 | 1160 | 2090 | 2.7 | 960 | 1549 | 1755 | 1.8 |
| Phosphodiesterase 4D, cAMP specific | Pde4d | ST | 54 | 49 | 145 | 2.7 | 50 | 29 | 114 | 2.3 |
| Chondrolectin | Chodl | Unk | 270 | 254 | 706 | 2.6 | 210 | 268 | 577 | 2.7 |
| Prolactin receptor | prlr |  | 29 | 25 | 74 | 2.5 | 24 | 24 | 88 | 3.7 |
| Fibronectin leucine rich transmembrane protein 3 | Flrt3 |  | 200 | 286 | 495 | 2.5 | 249 | 223 | 499 | 2.0 |
| Suppression of tumorigenicity 18 | St18 |  | 265 | 335 | 643 | 2.4 | 211 | 281 | 521 | 2.5 |
| Zinc finger protein, multitype 2 | Zfpm2 | TX | 144 | 167 | 347 | 2.4 | 160 | 155 | 329 | 2.1 |
| Leucine rich repeat transmembrane neuronal 4 | Lrrtm4 |  | 58 | 50 | 140 | 2.4 | 57 | 26 | 92 | 1.6 |
| HECT domain containing 2 | Hectd2 |  | 40 | 58 | 96 | 2.4 | 53 | 74 | 69 | 1.3 |
| Immunoglobin superfamily, member 21 | Igsf21 |  | 488 | 870 | 1160 | 2.4 | 417 | 640 | 1127 | 2.7 |
| Homeo box A5 | Hoxa5 | TX | 628 | 896 | 1493 | 2.4 | 826 | 796 | 1026 | 1.2 |
| Neurogenic differentiation 1* | Neurod1^2^ | TX | 1048 | 1468 | 2440 | 2.3 | 1352 | 1190 | 2481 | 1.8 |
| CXXC finger 4 | Cxxc4^2^ |  | 61 | 49 | 141 | 2.3 | 47 | 41 | 75 | 1.6 |
| p21-activated kinase 3 | Pak3^2^ | SY | 246 | 270 | 568 | 2.3 | 308 | 378 | 605 | 2.0 |
| Semaphorin 3D | Sema3d | AX | 318 | 365 | 725 | 2.3 | 513 | 324 | 631 | 1.2 |
| Protocadherin 17 | Pcdh17^2^ | AX | 331 | 340 | 746 | 2.3 | 272 | 235 | 673 | 2.5 |
| Mab-21-like 1 | Mab21l1 |  | 95 | 31 | 214 | 2.3 | 26 | 36 | 165 | 6.4 |
| Cortactin binding protein 2 | Cttnbp2 |  | 52 | 67 | 117 | 2.3 | 27 | 28 | 112 | 4.1 |
| Spondin 1, (f-spondin) | Spon1 | AX | 629 | 778 | 1413 | 2.2 | 658 | 814 | 1214 | 1.8 |
| zinc finger protein 503 | Zfp503^2^ |  | 182 | 155 | 397 | 2.2 | 174 | 152 | 319 | 1.8 |
| potassium voltage-gated channel, Isk-related family, member 1-like | Kcne1l | NT | 613 | 813 | 1328 | 2.2 | 844 | 779 | 1024 | 1.2 |
| Deleted in colorectal carcinoma | Dcc | AX | 93 | 101 | 200 | 2.2 | 103 | 64 | 191 | 1.8 |
| Prickle-like 2 | Prickle2^3^ |  | 195 | 339 | 412 | 2.1 | 252 | 196 | 448 | 1.8 |
| Netrin G1 | Ntng1 | AX | 388 | 556 | 812 | 2.1 | 474 | 468 | 892 | 1.9 |
| Neuropilin 2* | Nrp2 | AX | 509 | 485 | 1063 | 2.1 | 408 | 404 | 1015 | 2.5 |
| Cadherin 22 | Cdh22 | AX | 199 | 226 | 414 | 2.1 | 239 | 283 | 423 | 1.8 |
| Synapsin II | Syn2^4^ | SY | 643 | 688 | 1324 | 2.1 | 720 | 980 | 1449 | 2.0 |
| Glutamate receptor, ionotropic, kainate 1 | Grik1 | NT | 221 | 241 | 455 | 2.1 | 187 | 239 | 406 | 2.2 |
| Protocadherin 8 | Pcdh8^2^ | AX | 278 | 398 | 569 | 2.0 | 232 | 272 | 529 | 2.3 |
| GDNF receptor alpha 2 | Gfra2 | Dev | 363 | 436 | 735 | 2.0 | 367 | 369 | 718 | 2.0 |
| Nescient helix loop helix 2, Nscl2 | Nhlh2 | TX | 1222 | 1996 | 2446 | 2.0 | 1251 | 1453 | 2366 | 1.9 |

**2.2 Decreased transcripts in E13.5 DRG of Brn3a knockout mice.**

|  |  |  | **Experiment** | | | | **Replicate** | | | |
| --- | --- | --- | --- | --- | --- | --- | --- | --- | --- | --- |
| **Gene Title** | **Symbol** | **Class** | **WT** | **HT** | **KO** | **WT/KO** | **WT** | **HT** | **KO** | **WT/KO** |
| Limb expression 1 | Lix1 | Unk | 984 | 923 | 46 | 21.4 | 841 | 721 | 86 | 9.8 |
| Vascular endothelial growth factor C | Vegfc | Dev | 55 | 54 | 3 | 16.1 | 85 | 54 | 40 | 2.1 |
| Neural cell adhesion molecule 2 | Ncam2^2^ | AX | 280 | 212 | 22 | 12.9 | 305 | 234 | 42 | 7.4 |
| Runt related 3 | Runx3 | TX | 251 | 421 | 22 | 11.3 | 543 | 439 | 112 | 4.9 |
| Brn3b | Pou4f2 | TX | 613 | 772 | 61 | 10.0 | 681 | 844 | 225 | 3.0 |
| Brn3a | Pou4f1^2^ | TX | 1107 | 1119 | 115 | 9.6 | 1304 | 924 | 328 | 4.0 |
| Phospholipase A2, group VII* | Pla2g7 | ST | 618 | 438 | 77 | 8.1 | 621 | 383 | 230 | 2.7 |
| K+ channel, shaker-related, member 1 | Kcna1^3^ | NT | 438 | 523 | 59 | 7.4 | 574 | 533 | 426 | 1.3 |
| Advillin* | Avil | AX | 2494 | 1735 | 364 | 6.9 | 2069 | 2483 | 825 | 2.5 |
| Galanin* | Gal | NT | 5388 | 4414 | 824 | 6.5 | 5784 | 5034 | 1898 | 3.0 |
| Basonuclin 1* | Bnc1 | TX | 875 | 740 | 165 | 5.3 | 912 | 712 | 266 | 3.4 |
| Insulin-like growth factor 1* | Igf1^3^ | Dev | 820 | 609 | 160 | 5.1 | 730 | 558 | 290 | 2.5 |
| G protein-coupled receptor 64* | Gpr64^2^ | NT | 371 | 298 | 72 | 5.1 | 391 | 427 | 136 | 2.9 |
| Adenylate cyclase activating polypeptide 1, PACAP* | Adcyap1 | NT | 283 | 198 | 57 | 5.0 | 236 | 214 | 74 | 3.2 |
| Regulator of G-protein signalling 10* | Rgs10 | NT | 1793 | 1596 | 362 | 5.0 | 1745 | 1545 | 589 | 3.0 |
| Parvalbumin | Pvalb | ST | 403 | 223 | 95 | 4.3 | 330 | 339 | 252 | 1.3 |
| K+ channel, shaker-related, beta member 2* | Kcnab2 | NT | 952 | 684 | 238 | 4.0 | 932 | 840 | 324 | 2.9 |
| Diacylglycerol kinase, eta | Dgkh^2^ | ST | 1348 | 1230 | 353 | 3.8 | 937 | 981 | 411 | 2.3 |
| G protein-coupled receptor 73 | Gpr73 | NT | 336 | 157 | 90 | 3.7 | 194 | 265 | 143 | 1.4 |
| Serine proteinase inhibitor, clade A, member 3G; Spi2A | Serpina3g | Other | 426 | 346 | 116 | 3.7 | 380 | 311 | 146 | 2.6 |
| Copine IV | Cpne4 | Unk | 767 | 636 | 216 | 3.6 | 685 | 637 | 303 | 2.3 |
| PQ loop repeat containing 1 | Pqlc1 | Unk | 911 | 596 | 259 | 3.5 | 657 | 588 | 456 | 1.4 |
| Reticulon 4 receptor-like 2; nogo receptor-like 3 | Rtn4rl2 | AX | 789 | 694 | 226 | 3.5 | 774 | 744 | 277 | 2.8 |
| Spermatogenesis associated glutamate-rich protein 1 | Speer1-ps1 | Unk | 349 | 307 | 106 | 3.3 | 313 | 308 | 144 | 2.2 |
| Pappalysin 2 | Pappa2 | Other | 694 | 794 | 225 | 3.1 | 972 | 1038 | 407 | 2.4 |
| Brn3c | Pou4f3 | TX | 394 | 361 | 130 | 3.0 | 408 | 345 | 224 | 1.8 |
| Protein tyrosine phosphatase, receptor type, J | Ptprj | ST | 853 | 918 | 284 | 3.0 | 1057 | 770 | 301 | 3.5 |
| Heparan sulfate 3-O-sulfotransferase 2; 3Ost2 | Hs3st2^2^ | Other | 779 | 666 | 260 | 3.0 | 740 | 688 | 344 | 2.2 |
| Popeye domain containing 3 | Popdc3 | Other | 219 | 165 | 73 | 3.0 | 132 | 197 | 87 | 1.5 |
| Docking protein 4* | Dok4 | ST | 2014 | 1655 | 679 | 3.0 | 1909 | 1738 | 1087 | 1.8 |
| Transgelin 2 | Tagln2 |  | 1841 | 1350 | 632 | 2.9 | 1586 | 1616 | 1037 | 1.5 |
| Kin of IRRE like 3 | Kirrel3 |  | 459 | 441 | 169 | 2.7 | 470 | 457 | 198 | 2.4 |
| Chromodomain helicase DNA binding protein 5 | Chd5 |  | 772 | 671 | 288 | 2.7 | 558 | 598 | 384 | 1.5 |
| TGF-beta induced | Tgfbi |  | 418 | 282 | 156 | 2.7 | 367 | 306 | 321 | 1.1 |
| Eph receptor A7 | Epha7 | AX | 206 | 202 | 77 | 2.7 | 152 | 160 | 95 | 1.6 |
| Latexin* | Lxn | NT? | 2948 | 2564 | 1105 | 2.7 | 2105 | 3016 | 1383 | 1.5 |
| DRG11 | Prrxl1 | TX | 1514 | 1339 | 569 | 2.7 | 1597 | 1158 | 771 | 2.1 |
| Protein tyrosine phosphatase, non-receptor type 3 | Ptpn3 | ST | 1638 | 1881 | 623 | 2.6 | 1775 | 1729 | 923 | 1.9 |
| GABA-A receptor, subunit alpha 2 | Gabra2 | NT | 62 | 90 | 24 | 2.6 | 72 | 64 | 69 | 1.1 |
| Src homology 2 domain C1* | Shc1 |  | 378 | 439 | 144 | 2.6 | 299 | 288 | 207 | 1.4 |
| Matrix gamma-carboxyglutamate protein | Mglap |  | 217 | 102 | 84 | 2.6 | 171 | 129 | 55 | 3.1 |
| Kelch-like 5 | Klhl5^2^ |  | 325 | 266 | 126 | 2.6 | 322 | 245 | 151 | 2.1 |
| ATPase, Na+/K+ transporting, alpha 1 polypeptide | Atp1a1 |  | 1194 | 898 | 480 | 2.5 | 1242 | 952 | 731 | 1.7 |
| Autism susceptibility candidate 2 | Auts2 |  | 828 | 936 | 337 | 2.5 | 1112 | 541 | 575 | 1.9 |
| Rho GTPase activating protein 26 | Arhgap26 |  | 337 | 276 | 137 | 2.5 | 350 | 254 | 205 | 1.7 |
| Human immunodeficiency virus type I enhancer binding protein 2 | Hivep2 |  | 827 | 641 | 345 | 2.4 | 680 | 671 | 393 | 1.7 |
| Na+ channel, type VII, alpha | Scn7a | NT | 1199 | 1386 | 506 | 2.4 | 1159 | 879 | 598 | 1.9 |
| Poliovirus receptor-related 3 | Pvrl3^2^ |  | 756 | 609 | 322 | 2.3 | 637 | 604 | 332 | 1.9 |
| ring finger protein 180 | Rnf180 |  | 267 | 179 | 114 | 2.3 | 292 | 179 | 129 | 2.3 |
| Protein tyrosine phosphatase, receptor type, R | Ptprr | ST | 3376 | 3207 | 1467 | 2.3 | 3696 | 3181 | 1724 | 2.1 |
| Rap1, GTPase-activating protein 1 | Rap1ga1 | ST | 1721 | 1686 | 749 | 2.3 | 1804 | 1809 | 889 | 2.0 |
| LIM domain binding 2 | Ldb2 | TX | 794 | 492 | 349 | 2.3 | 599 | 515 | 469 | 1.3 |
| Anthrax toxin receptor 2 | Antxr2 |  | 1355 | 1095 | 608 | 2.2 | 1209 | 1123 | 776 | 1.6 |
| Runt related transcription factor 1* | Runx1 | TX | 360 | 343 | 162 | 2.2 | 387 | 292 | 263 | 1.5 |
| G protein-coupled receptor, family C, group 5, member C | Gprc5c | NT | 273 | 241 | 125 | 2.2 | 286 | 286 | 128 | 2.2 |
| FK506 binding protein 1b* | Fkbp1b |  | 3969 | 3594 | 1832 | 2.2 | 3683 | 3887 | 2016 | 1.8 |
| Homeo box D1* | Hoxd1 | TX | 852 | 751 | 404 | 2.1 | 669 | 662 | 369 | 1.8 |
| Pleckstrin homology, Sec7 and coiled-coil domains 3 | Pscd3 |  | 2090 | 1319 | 996 | 2.1 | 1906 | 2147 | 1160 | 1.6 |
| F-box only protein 9 | Fbxo9 |  | 300 | 230 | 147 | 2.0 | 361 | 311 | 206 | 1.8 |
| Receptor (calcitonin) activity modifying protein 3 | Ramp3 |  | 423 | 739 | 209 | 2.0 | 943 | 876 | 472 | 2.0 |
| Basonuclin 2 | Bnc2 |  | 2610 | 1857 | 1300 | 2.0 | 2406 | 2230 | 1546 | 1.6 |
| Microtubule-associated protein 7 | Mtap7 |  | 376 | 384 | 188 | 2.0 | 436 | 374 | 279 | 1.6 |
| Inhibitor of DNA binding 1* | Id1 | TX | 1248 | 1051 | 624 | 2.0 | 1220 | 1243 | 785 | 1.6 |
