# Supplementary material for: POU-domain factor Brn3a regulates both distinct and common programs of gene expression in the spinal and trigeminal sensory ganglia
Source: Neural Dev. 2007 Jan 19;2:3. doi: 10.1186/1749-8104-2-3 (PMC1796875; doi:10.1186/1749-8104-2-3)
Supplement: Additional file 3 — Direct comparision of altered gene expression in DRG and TG of Brn3a knockout sensory ganglia. Gene expression in the DRG is analyzed using the U74v2 array set to allow direct comparision to a prior data set for the TG. [file 1749-8104-2-3-S3.doc]

**Additional file 3**

**Direct comparision of altered gene expression in DRG and TG of Brn3a knockout sensory ganglia**

**3.1. Ranked by transcripts most increased in the DRG.**

Fold change represents the ratio of knockout/wildtype expression for each trancript. Tables S3-S6 are the result of a comparision between the DRG using Affymetrix U74Av2 and U74Bv2 arrays and previously reported data for the trigeminal using this array set (Eng, et al., 2004). Progress in the annotation of the mouse genome has allowed the identification of many transcripts which were not annoated at the time of the original study.

| **Increased transcripts in DRG** | | | |
| --- | --- | --- | --- |
| **Gene Title** | **Symbol** | **DRG**  **Fold** | **TG fold** |
| Somatostatin | Sst | **13.13** | **29.1** |
| Semaphorin 3C 101178_at | Sema3c | 10.31 | abs |
| Cell adhesion molecule with homology to L1CAM | Chl1 | **9.29** | **13.7** |
| Neurogenic differentiation 6 | Neurod6 | 8.28 | (1.5) |
| C-fos induced growth factor | Figf | **7.27** | **12.7** |
| Leucine rich repeat containing 4B | Lrrc4b | 6.91 | (2.0) |
| Musculin | Msc | **5.35** | **8.8** |
| Secretogranin II | Scg2 | **5.08** | **2.9** |
| Disabled homolog 1 | Dab1 | **4.65** | **3.7** |
| G protein-coupled receptor 160 | Gpr160 | 3.46 | abs |
| Follistatin-like 5 | Fstl5 | **3.01** | **4.7** |
| GABA transporter | Slc6a1 | **2.99** | **2.2** |
| Cholecystokinin A receptor | Cckar | **2.91** | **2.3** |
| Neurogenic differentiation 1 | Neurod1 | **2.84** | **3.3** |
| Guanylate cyclase 1, soluble, alpha 3 | Gucy1a3 | 2.73 | abs |
| Homeo box A5 | Hoxa5 | 2.72 | abs |
| EGF-like repeats and discoidin I-like domains 3 | Edil3 | 2.71 | abs |
| Spondin 1, (f-spondin) | Spon1 | **2.57** | **1.7** |
| Growth arrest and DNA-damage-inducible 45 alpha | Gadd45a | **2.57** | **2.2** |
| Mus musculus zinc finger homeobox 1b | Zfhx1b | 2.55 | abs |
| Nel-like 2 homolog | Nell2 | 2.51 | (2.4) |
| Insulinoma-associated 1 | Insm1 | 2.43 | abs |
| K+ channel, Isk-related family, member 1-like | Kcne1l | **2.37** | **2.1** |
| TRP cation channel, subfamily C, member 4 | Trpc4 | 2.35 | NC |
| Protein kinase, cGMP-dependent, type II | Prkg2 | 2.27 | NC |
| Bruno-like 4, RNA binding protein | Brunol4 | **2.25** | **2.7** |
| Cytochrome P450, 1b1 | Cyp1b1 | **2.15** | **3.0** |
| Fibronectin leucine rich transmembrane protein 3 | Flrt3 | 2.13 | NC |
| Aldehyde dehydrogenase family 1, subfamily A2 | Aldh1a2 | 2.13 | abs |
| Homeo box C4 | Hoxc4 | 2.12 | abs |
| Short stature homeobox 2 | Shox2 | **2.08** | **2.6** |
| GDNF receptor alpha 1 | Gfra1 | 2.08 | abs |
| Peroxin 2 | Pex2 | 2.04 | NC |
| Glutamate receptor, kainate 1 | Grik1 | 2.03 | NC |
| EGF receptor pathway substrate 15 | Eps15 | 2.03 | NC |
| Cadherin 6 | Cdh6 | 2.01 | NC |
| Plexin A2 | Plxna2 | 2.01 | NC |

Legend for Tables S3-S6: NC, no change; abs, absent call. Numerical values are shown only in case where change P value was significant (p<0.003 (increased) or P>0.997 (decreased). Figures in parentheses indicate that change P was significant, but present/absent call was not. Concordant changes appear in bold face.

**3.2.**  **Ranked by transcripts most decreasd in the DRG.**  Fold change represents the

| **Decreased transcripts in DRG** | | | |
| --- | --- | --- | --- |
| **Gene Title** | **Gene Symbol** | **DRG**  **fold** | **TG fold** |
| K+ channel, shaker-related, member 1 | Kcna1 | 12.25 | (8.4) |
| Regulator of G-protein signalling 10 | Rgs10 | **10.76** | **11.0** |
| Insulin-like growth factor 1 | Igf1 | **10.26** | **3.8** |
| Neural cell adhesion molecule 2 | Ncam2 | 9.98 | abs |
| Blood vessel epicardial substance | Bves | **9.44** | **7.4** |
| Spermatogenesis glutamate-rich protein 1 | Speer1-ps1 | 9.15 | abs |
| Phospholipase A2, group VII | Pla2g7 | **7.44** | **6.5** |
| Secreted phosphoprotein 1 | Spp1 | 7.39 | abs |
| Basonuclin 1 | Bnc1 | **7.24** | **9.0** |
| Advillin | Avil | **6.44** | **10.3** |
| Galanin | Gal | **5.04** | **4.0** |
| Popeye domain containing 3 | Popdc3 | **4.91** | **8.1** |
| Oxysterol binding protein-like 3 | Osbpl3 | **4.88** | **3.2** |
| K+ channel, shaker-related, beta member 2 | Kcnab2 | **4.42** | **7.9** |
| Src homology 2 domain-containing C1 | Shc1 | 3.84 | (4.3) |
| Brn3a | Pou4f1 | **3.78** | **6.0** |
| Diacylglycerol kinase, eta | Dgkh | 3.65 | (2.9) |
| Adenylate cyclase activating polypeptide 1 | Adcyap1 | **3.57** | **5.6** |
| Mitochondrial tumor suppressor 1 | Mtus1 | **3.30** | **1.9** |
| Wiskott-Aldrich syndrome protein interacting protein | Waspip | **3.13** | **4.2** |
| Docking protein 4 | Dok4 | **3.12** | **5.2** |
| Limb expression 1 homolog | Lix1 | **3.10** | **1.9** |
| Na+, voltage-gated, type VII, alpha | Scn7a | **2.95** | **3.0** |
| Sialyltransferase | St8sia4 | **2.82** | **1.6** |
| Dedicator of cytokinesis 5 | Dock5 | **2.72** | **3.4** |
| Protein tyrosine phosphatase, receptor type, R | Ptprr | **2.54** | **1.4** |
| G protein-coupled receptor 64 | Gpr64 | **2.53** | **3.4** |
| Protein tyrosine phosphatase, non-receptor type 3 | Ptpn3 | **2.49** | **5.1** |
| Serine proteinase inhibitor, clade A, member 3G | Serpina3g | 2.43 | (2.3) |
| GABA-A receptor, subunit alpha 2 | Gabra2 | 2.41 | (2.2) |
| Reticulon 4 receptor-like 1 | Rtn4rl1 | **2.40** | **2.9** |
| Beta-2 microglobulin | B2m | 2.38 | nc |
| ATP-binding cassette, sub-family C, member 4 | Abcc4 | **2.37** | **1.8** |
| Fizzy/cell division cycle 20 related 1 | Fzr1 | **2.33** | **3.0** |
| transgelin 2 | Tagln2 | **2.32** | **2.0** |
| Na+ channel, voltage-gated, type IX, alpha | Scn9a | **2.32** | **3.2** |
| Immunoglobulin superfamily, member 4 | Igsf4d | **2.27** | **2.6** |
| Poliovirus receptor-related 3 | Pvrl3 | 2.25 | nc |
| RAS p21 protein activator 4 | Rasa4 | **2.25** | **5.6** |
| Parvalbumin | Pvalb | 2.22 | abs |
| Homeo box D1 | Hoxd1 | **2.20** | **8.0** |
| Latexin | Lxn | **2.20** | **2.9** |
| Sorting nexin 7 | Snx7 | 2.15 | nc |
| HIV type I enhancer binding protein 2 | Hivep2 | **2.15** | **3.3** |
| Protein phosphatase 2, subunit B, gamma isoform | Ppp2r2c | **2.06** | **2.0** |
| Actin-binding LIM protein 1 | Ablim1 | **2.05** | **2.8** |
| Anthrax toxin receptor 2 | Antxr2 | **2.03** | **2.4** |
| Desmuslin | Dmn | **2.01** | **2.5** |
| SERTA domain containing 4 | Sertad4 | **2.00** | **1.3** |

ratio of widltype/knockout expression for each trancript.

**3.3. Ranked by transcripts most increased in the TG.** Fold change represents the ratio of knockout/wildtype expression for each transcript.

| **Increased transcripts in trigeminal** | | | |
| --- | --- | --- | --- |
| **Gene Title** | **Symbol** | **TG**  **Fold** | **DRG fold** |
| GATA binding protein 3 | Gata3**^2^** | 109.8 | (10.1) |
| Transcription factor AP-2 beta | Tcfap2b^2^ | 38.8 | abs |
| Somatostatin | Sst | **29.0** | **13.1** |
| Ankyrin repeat domain 1 | Ankrd1 | 22.4 | abs |
| Calbindin 2 (Calretinin) | Calb2 | 22.2 | abs |
| Close homolog of L1 | Chl1**^2^** | **13.6** | **9.3** |
| C-fos induced growth factor | Figf | **12.7** | **7.3** |
| RAB3B, member RAS oncogene family | Rab3b | 11.2 | abs |
| Musculin, MyoR | Msc | **8.8** | **5.4** |
| LIM and cysteine-rich domains 1 | Lmcd1 | 7.6 | abs |
| Neurogenic differentiation 4, Math3 | Neurod4^2^ | 6.6 | nc |
| Zinc finger and BTB domain containing 16 | Zbtb16 | **6.6** | **2.0** |
| Iroquois related homeobox 1 | Irx1^2^ | **6.0** | **2.6** |
| 5-HT (serotonin) receptor 3A | Htr3a | **5.6** | **1.8** |
| Transmembrane protein 46 | Tmem46 | **5.3** | **2.4** |
| Iroquois related homeobox 2 | Irx2 | 4.6 | abs |
| Follistatin-like 5 | Fstl5 | **4.6** | **3.0** |
| Protocadherin 17 | Pcdh17^2^ | **4.3** | **1.3** |
| Gap junction channel alpha 1 | Gja1^2^ | 4.3 | nc |
| Neuropilin 2 | Nrp2 | **3.9** | **1.9** |
| Thrombospondin 1 | Thbs1 | 3.8 | abs |
| Disabled homolog 1 | Dab1^2^ | 3.7 | (4.7) |
| Bruno-like 4, RNA binding protein | Brunol4^3^ | **3.5** | **1.9** |
| Neurogenic differentiation 1 | Neurod1 | **3.3** | **2.0** |
| Natriuretic peptide precursor type B | Nppb | 3.3 | nc |
| Zinc finger homeobox 1b | Zfhx1b^2^ | **3.2** | **1.8** |
| Cytochrome P450 1b1 | Cyp1b1 | **2.9** | **2.2** |
| Glial high affinity glutamate transporter | Slc1a3 | 2.9 | nc |
| Serine proteinase inhibitor, clade I, member 1 | Serpini1 | **2.9** | **1.5** |
| Eyes absent 2 homolog | Eya2 | **2.8** | **1.5** |
| Semaphorin 3C 110193_at | Sema3c | **2.8** | **2.0** |
| Nnucleosome assembly protein 1-like 5 | Nap1l5 | **2.8** | **1.2** |
| Ly6/Plaur domain containing 1 | Lypdc1 | **2.8** | **2.5** |
| Nuclear receptor COUP-TF2 | Nr2f2 | 2.8 | nc |
| Cytoglobin | Cygb^3^ | 2.7 | nc |
| ADP-ribosyltransferase 3 | Art3 | 2.7 | nc |
| Alpha-2-macroglobulin | A2m | 2.7 | abs |
| Galectin-7 | Lgals7 | 2.6 | abs |
| Coactosin-like 1 | Cotl1 | **2.6** | **1.4** |
| Short stature homeobox 2 | Shox2 | **2.6** | **2.1** |
| Cortactin binding protein 2 | Cttnbp2 | **2.5** | **1.9** |
| Glutamate receptor, ionotropic, AMPA3 | Gria3 | **2.5** | **1.9** |
| LIM domain only 4 | Lmo4 | 2.5 | nc |
| Nel-like 2 | Nell2 | **2.5** | **2.2** |
| Sorbin and SH3 domain containing 1 | Sorbs1 | 2.5 | nc |
| **Selected:** |  |  |  |
| Ets variant gene 1, ER81 | Etv1 | 2.1 | d 0.8 |
| Tyrosine kinase receptor trkB | Ntrk2 | 2.0 | nc |

**3.4. Ranked by transcripts most decreased in the TG.** Fold change represents the ratio of wildtype/knockout expression for each transcript.

| **Decreased transcripts in trigeminal** | | | |
| --- | --- | --- | --- |
| **Gene Title** | **Gene Symbol** | **TG**  **fold** | **DRG fold** |
| Neuropeptide Y receptor Y1 | Npy1r | 32.0 | abs |
| Fanconi anemia, complementation group C | Fancc | **15.6** | **1.9** |
| Regulator of G-protein signalling 10 | Rgs10^2^ | **14.0** | **4.6** |
| Advillin | Avil | **10.4** | **6.4** |
| Basonuclin 1 | Bnc1 | **9.0** | **7.2** |
| Homeo box D1 | Hoxd1 | **8.0** | **2.2** |
| K+ channel, shaker-related subfamily beta 2 | Kcnab2^2^ | **7.9** | **4.4** |
| Na+ channel, voltage-gated, type VII, alpha | Scn7a^2^ | **7.8** | **2.7** |
| DIRAS family, GTP-binding RAS-like 2 | Diras2 | **7.8** | **2.3** |
| Blood vessel epicardial substance | Bves | **7.4** | **9.4** |
| H6 homeo box 1 | Hmx1 | **7.2** | **1.3** |
| Phospholipase A2, group VII | Pla2g7 | **6.5** | **7.4** |
| Brn3a | Pou4f1 | **6.0** | **3.8** |
| Adenylate cyclase activating polypeptide 1, PACAP | Adcyap1 | **5.6** | **3.6** |
| K+ channel, shaker-related subfamily 1 | Kcna1 | **5.6** | **1.8** |
| RAS p21 protein activator 4 | Rasa4 | **5.6** | **2.6** |
| Ret proto-oncogene | Ret | 5.4 | **↑**0.5 |
| Insulin-like growth factor 1 | Igf1 | **5.2** | **3.1** |
| Docking protein 4 | Dok4^2^ | **5.2** | **3.1** |
| Protein tyrosine phosphatase, non-receptor type 3 | Ptpn3 | **5.2** | **2.5** |
| Diacylglycerol kinase, eta | Dgkh | **5.1** | **2.7** |
| Solute (anion) carrier family 4, member 4 | Slc4a4^3^ | **5.1** | **1.6** |
| Runt related transcription factor 1 | Runx1^2^ | **5.0** | **2.0** |
| Chimerin 2 | Chn2^2^ | 4.6 | abs |
| Eph receptor A7 | Epha7 | 4.3 | abs |
| WASP interacting protein | Waspip | **4.2** | **3.1** |
| Galanin | Gal | **4.0** | **5.0** |
| Synaptotagmin-like 2 | Sytl2 | **4.0** | **2.5** |
| Male sterility domain containing 1 | Mlstd1 | **4.0** | **2.3** |
| ShcA, p66Shc | Shc1 | **3.9** | **3.7** |
| Olfactomedin 1 | Olfm1 | **3.9** | **1.9** |
| Transmembrane and coiled coil domains 3 | Tmcc3 | 3.7 | nc |
| T-box 3 | Tbx3 | 3.6 | nc |
| Ataxin 2 binding protein 1 | A2bp1^2^ | **3.6** | **1.4** |
| Ras homolog gene family, member U | Rhou | **3.5** | **1.4** |
| G protein-coupled receptor 64 | Gpr64 | **3.5** | **2.5** |
| Dedicator of cytokinesis 5 | Dock5 | **3.4** | **2.7** |
| Rap guanine nucleotide exchange factor (GEF) 4 | Rapgef4 | 3.3 | nc |
| Phosphofructokinase, platelet | Pfkp | 3.3 | nc |
| HIV type I enhancer binding protein 2 | Hivep2^2^ | **3.3** | **2.2** |
| Leucine rich repeat and fibronectin type III domain 5 | Lrfn5^2^ | **3.3** | **1.4** |
| Oxysterol binding protein-like 3 | Osbpl3 | **3.2** | **4.9** |
| Sodium channel, voltage-gated, type IX, alpha | Scn9a | **3.2** | **2.3** |
| Ring finger protein 125 | Rnf125 | **3.0** | **2.8** |
| **Selected:** |  |  |  |
| Reticulon 4 receptor-like 1, Nogo-66 receptor 2 | Rtn4rl1 | **2.9** | **2.4** |
| Latexin | Lxn | **2.9** | **2.2** |
| Actin-binding LIM protein 1 | Ablim1^2^ | **2.8** | **2.1** |
| Inhibitor of DNA binding 1 | Id1 | **2.5** | **2.0** |
| Nerve growth factor receptor | Ngfr | **2.5** | **1.9** |
| Neuropilin 1 | Nrp1 | **2.3** | **1.4** |
