# Supplementary material for: POU-domain factor Brn3a regulates both distinct and common programs of gene expression in the spinal and trigeminal sensory ganglia
Source: Neural Dev. 2007 Jan 19;2:3. doi: 10.1186/1749-8104-2-3 (PMC1796875; doi:10.1186/1749-8104-2-3)
Supplement: Additional file 4 — Dorsal root ganglia exhibit significant compensation for the loss of one Brn3a allele. Gene expression levels are compared for Brn3a wild-type, heterozygote, and knockout ganglia to demonstrate the extent to which gene dosage compensation reduces the heterozygote phenotype. [file 1749-8104-2-3-S4.doc]

**Additional file 4**

**Dorsal root ganglia exhibit significant compensation for the loss of one Brn3a allele.** Global gene expression was assayed in Brn3a wildtype, heterozygote and knockout E13.5 DRG using 430A+B arrays. Quantitative data from a single comparision were used in the analysis (Table 2, 3), but only transcripts with statistically significant changes (p<0.005, p>0.995) in a replicate analysis were included. Duplicate probe sets and unidentified transcripts were excluded from the data set. (A) Heterozygote phenotype for the 20 genes showing the greatest increase in knockout ganglia, mean fold change 5.2, range 3-27 fold. (B) Heterozygote phenotype for 34 moderately increased genes, mean fold change 2.3, range 2-3 fold. (C) Heterozygote phenotype for the 31 most decreased genes, mean fold change 6.3, range 3-21 fold. (D) Heterozygote phenotype for 33 moderately decreased genes, mean fold change 2.4, range 2-3 fold. In each case, gene expression in the heterozygous ganglia is either increased or decreased by 16-19% of the change observed in knockout ganglia, compared to the 50% change that would be expected if the expression levels from the two alleles were completely independent.

**
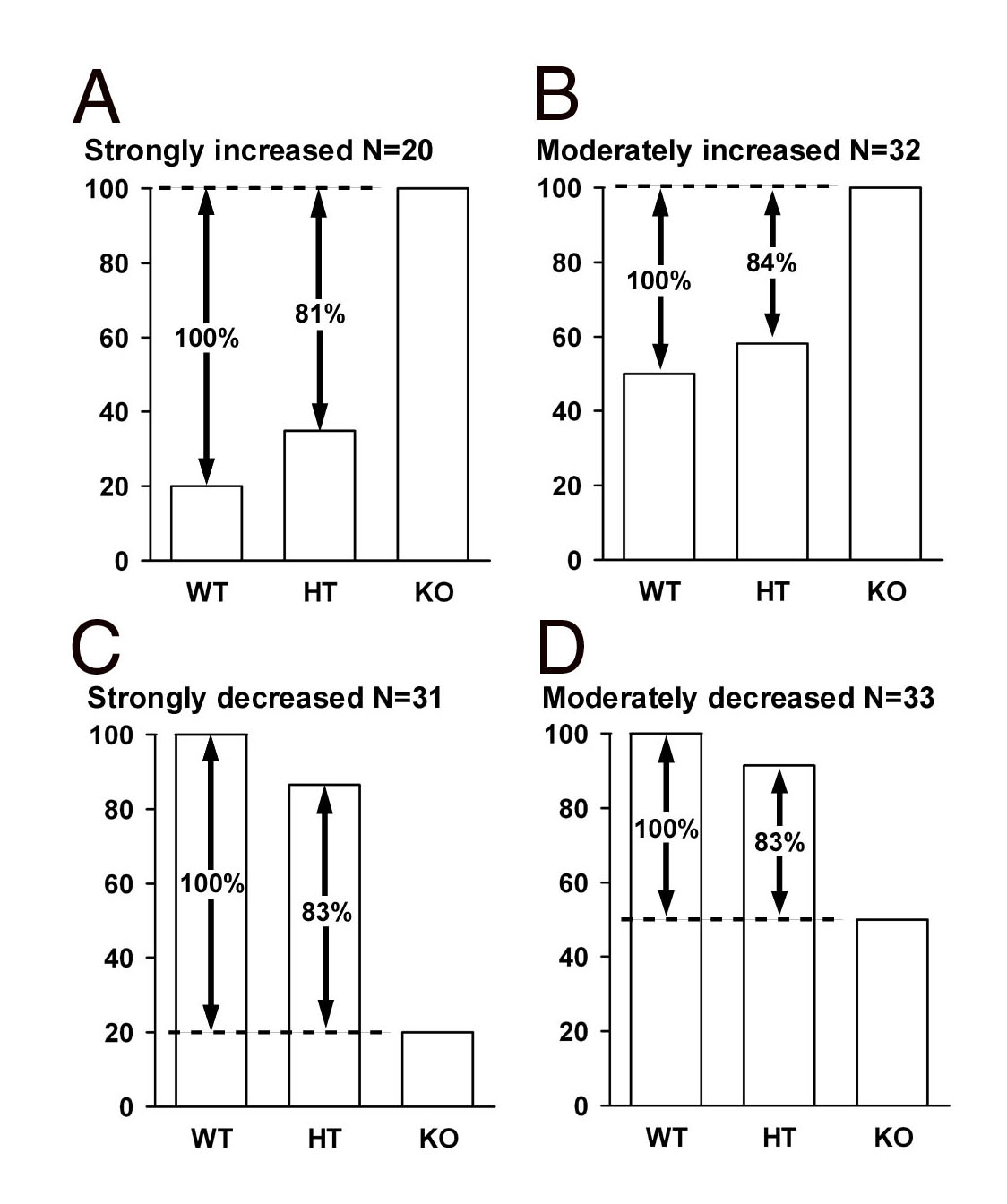
**
