# Supplementary material for: POU-domain factor Brn3a regulates both distinct and common programs of gene expression in the spinal and trigeminal sensory ganglia
Source: Neural Dev. 2007 Jan 19;2:3. doi: 10.1186/1749-8104-2-3 (PMC1796875; doi:10.1186/1749-8104-2-3)
Supplement: Additional file 5 — Summary of insitu hybridization probes andLocus-ChIP oligonucleotides. Summary of insitu hybridization probes andLocus-ChIP oligonucleotides. [file 1749-8104-2-3-S5.doc]

**Additional File 5**

**5.1 Summary of insitu hybridization probes.**

| **A. Probes obtained from other laboratories** | |
| --- | --- |
| **Gene** | **Reference** |
| RGS4 | (Grillet et al., 2003) |
| Sostdc1 (ectodin) | (Laurikkala et al., 2003) |
| Msc, MyoR | (Lu et al., 1999) |
| NeuroD1 | (Tomita et al., 2000) |
| Htr3a | (Zeitz et al., 2002) |
| NpyR1 | ATCC accession #10469632 |

| **B. Probes generated by PCR** | | |
| --- | --- | --- |
| **Probe** | **5’ oligonucleotide** | **3’ oligonucleotide** |
| Antrx2 | CGATAGCCCATGATTCACGACACT | CGTTGACGAGACACGATGGTGATA |
| Avil | CAGTCACAGAGGTGGCAACAAGGCCC | CTCCGCAGCAGAAAGACGTCGTTGG |
| Bnc | ACCTGGCAAGCTCTGAGACC | CTTTCGACTCTTCCAGAAAAGG |
| Dgkh | CATAGCGGTGTTGAACATCCCT | CGTGAGGAGATTCCAAGTGCA |
| Ednrb | ACACTCGCCGAATCTCACTGTCCT | CGAGAGAGCTGGTTACAGTGACTA |
| Gpr64 | ATGTCATTCTCGTTACATCCCTGAA | AAGCTTTCTCCATCTGGGATACTTG |
| Lgals7 | TGGAAAGGGAGGAGCTGAACCACT | TACAGCTACACTTTATTCGCCATC |
| Lix1 | CAAGGAGTTCATCATGGAGAGTGTT | TTCAATTTTCAGGGAGTTCATAGCA |
| Lxn | CCCAAGATGGAAATCCCACC | CGGTTGTCTACGGGTTAGCG |
| Pla2g7 | AGATCTGATGTTCGGTTATGGGAAT | AATCAGGTTCTCATCATCTCCTTCC |
| Ptprr | GATGGTTTGCATCAGAGTTTACCAC | GTGTGACTTCCTTGCTTTAAGACGA |
| Tbx3 | GGTCTCTCCATTCCAGTTTGGTCA | TGCAGCCAGCTCTACTTGAAAGCA |

**References for additional file 5.1**

**Grillet, N., Dubreuil, V., Dufour, H. D. and Brunet, J. F.** (2003). Dynamic expression of RGS4 in the developing nervous system and regulation by the neural type-specific transcription factor Phox2b. *J Neurosci* **23**, 10613-21.

**Laurikkala, J., Kassai, Y., Pakkasjarvi, L., Thesleff, I. and Itoh, N.** (2003). Identification of a secreted BMP antagonist, ectodin, integrating BMP, FGF, and SHH signals from the tooth enamel knot. *Dev Biol* **264**, 91-105.

**Lu, J., Webb, R., Richardson, J. A. and Olson, E. N.** (1999). MyoR: a muscle-restricted basic helix-loop-helix transcription factor that antagonizes the actions of MyoD. *Proc Natl Acad Sci U S A* **96**, 552-7.

**Tomita, K., Moriyoshi, K., Nakanishi, S., Guillemot, F. and Kageyama, R.** (2000). Mammalian achaete-scute and atonal homologs regulate neuronal versus glial fate determination in the central nervous system. *Embo J* **19**, 5460-72.

**Zeitz, K. P., Guy, N., Malmberg, A. B., Dirajlal, S., Martin, W. J., Sun, L., Bonhaus, D. W., Stucky, C. L., Julius, D. and Basbaum, A. I.** (2002). The 5-HT3 subtype of serotonin receptor contributes to nociceptive processing via a novel subset of myelinated and unmyelinated nociceptors. *J Neurosci* **22**, 1010-9.

**5.2. Locus-ChIP oligonucleotides.**  The position of each oligonucleotide pair is given relative to the transcription start site.

| **A. Tcfap2b oligonucleotides** | | |
| --- | --- | --- |
| Name | Position | Sequence (5'-3') |
| AP2B-30 F | -9904 | GGAGCTGCCTTGAGATCTTCA |
| AP2B-30 R |  | GGCACGTACAGTGCTGGAGTT |
| AP2B-32 F | -7582 | CTGAGCTTCCTAGGTCCCATTGT |
| AP2B-32 R |  | ACTGGGCAGGATGCTTGC |
| AP2B-34 F | -5292 | ACTGGCACTGTGGACTACAGCTC |
| AP2B-34 R |  | AGCTTAACTATAAATTGGAAGCTGGG |
| AP2B-35 F | -4424 | CATGAGCAATAAACCTTGTGATGG |
| AP2B-35 R |  | AAAACCTTTGCATGGTGGACAT |
| AP2B-36b F | -3730 | AGCTGGCAGGAACAGTGCTT |
| AP2B-36b R |  | CACCATTTAAACTTTGGTCACCG |
| AP2B-36 F | -3095 | ATGCGTAACGGGAAACGTGT |
| AP2B-36 R |  | CATGCGCTTTGGGTAAGAGC |
| AP2B-37 F | -2614 | TCCTACAGAGGGCGGTGTTG |
| AP2B-37 R |  | AAATCCCAGCTATTTCGCCA |
| AP2-38B F | -1868 | TAAGTCCTTCCACACAACGACATT |
| AP2-38B R |  | CCCTTTCGCCACCAGAGAT |
| AP2B-38 F | -1435 | TCCTCAGCTTGGCTGTTAGGA |
| AP2B-38 R |  | GGAGGCTCCGTGAGTAGGAGA |
| AP2-39B F | -825 | TCACAAATTACAGGCAATAAACCC |
| AP2-39B R |  | ATGTTAATCTTGAGACCTTTAACAGGG |
| AP2B-39 F | -352 | GGACTGATGTGCGTGGTTTCT |
| AP2B-39 R |  | AATGGCGAATCAATGCAGC |
| AP2B-40b F | 80 | GAGCGTCGGATTTGGTGTGT |
| AP2B-40b R |  | TTCTCAGGACTCCGCTGTCTG |
| AP2-40c F | 545 | GAGCCAGGAATTCAGGCAAAC |
| AP2-40c R |  | CCCGATTCTGTCCCGTACTC |
| AP2-41b F | 1300 | GCAGAGGTGGACAACCCAGA |
| AP2-41b R |  | CAACACCTACAAGGCTCACGG |
| AP2B-41 F | 1724 | TACTGAGGAACCGCGCCTC |
| AP2B-41 R |  | GGAGTTGAACATAATCCCGCC |
| AP2B-42 F | 2215 | CCCAACGTGGTAGAAGGAGC |
| AP2B-42 R |  | GGCCTGACTGCCATTTCTGT |
| AP2-42b F | 2645 | CCCCACCAGATAATCAGGCA |
| AP2-42b R |  | GTAGTCTTGGGTGCTTGGTGTG |
| AP2B-43b F | 3161 | TGCAGAAGAAAAGAGGCCAAA |
| AP2B-43b R |  | GAGGTGCCTGTCACAGACAGG |
| AP2B-43 F | 3653 | AATTGGTAGAGGCCGCAGC |
| AP2B-43 R |  | CCCAGCTTACTCCAGGAGCTC |
| AP2B-44 F | 4429 | GCTGGGTAGTGGGTGAGCTTC |
| AP2B-44 R |  | GTCAATAGCTCCACACCGGG |
| AP2B-45 F | 5687 | GGAGAAGTTGGCACCAGGAG |
| AP2B-45 R |  | AGATAGCCAGCACTTTGCCC |
| AP2-46 F | 6592 | GCAAAACGTGGTGATTGTCAA |
| AP2-46 R |  | CAAGGTTCCTCTAAAACAATGAAGC |
| AP2B-47 F | 7300 | CCCTTAGCTGGTAGCCTGGC |
| AP2B-47 R |  | CTTCTCGAACTTAGGGCCCA |
| AP2B-48 F | 8521 | CGCACCAACATGGTTGTGA |
| AP2B-48 R |  | CCTGGGAGTGAAACCCATCA |
| AP2B-49 F | 9580 | CAGCGAGCAGCAAGAAGCT |
| AP2B-49 R |  | GCCTCCAGGACCTCACACC |
| AP2B-51 F | 11679 | CTCCCTCTTTGTGGCAAGCTA |
| AP2B-51 R |  | CTCTGCCAGCCAATTGATGTT |
| AP2B-52 F | 12717 | AGCAAACGAGTGGAGTGAAGG |
| AP2B-52 R |  | GCCGTAAACGAGCTCTGGAG |
| AP2B-53 F | 13569 | TTTTGCCCCAGGAGTCTGAG |
| AP2B-53 R |  | TGGAAAGGCAAAGTTATTGAGAGG |

| **B. Gata3 oligonucleotides** | | |
| --- | --- | --- |
| Name | Position | Sequence (5'-3') |
| dpGATA 97 F | -10239 | CACCAGGAGCAGGTCACATTT |
| dpGATA 97 R |  | AACCGCACATCAGAGGTGTGT |
| dpGATA 98 F | -8746 | CTTTGTGCAGCCCCTCACTC |
| dpGATA 98 R |  | AGCTTCTTGTTTAGAAATAACCTGCC |
| dpGATA 99 F | -7779 | GGCTAGGGTGATGTTGAATGC |
| dpGATA 99 R |  | ACCTCTGAGAGCACCTGGCA |
| dpGATA 100 F | -6839 | ACTTCCCAGTGGGTCACCC |
| dpGATA 100 R |  | TGTGCACTTTCTAGCTATCCGTG |
| dpGATA 101 F | -5571 | CGGCTTTTCTCCTCTCAAACA |
| dpGATA 101 R |  | TTTCTTGGAGCATGGGTTGG |
| GATA-102 F | -5000 | GATCGAGCTGGAATGGGAAG |
| GATA-102 R |  | TTAAATTCACAGCCCGGGAC |
| dpGATA 103 F | -4236 | TGTCTCAGCTTACTCCACGCA |
| dpGATA 103 R |  | GGTATGTGTGCCCTCTTGGG |
| GATA-103b F | -3425 | TGACGTCAAACCCAGTGTGG |
| GATA-103b R |  | GAAAGAGGACGTTGCTCTTTTGAT |
| dpGATA 104 F | -2704 | GGTTTCCGGTGAATGTTAGACAG |
| dpGATA 104 R |  | GCTGCCAGCATCTCTTCCAG |
| GATA-104b F | -2161 | GGATGACGCCTTCAGTTCCA |
| GATA-104b R |  | GGTGTTCCCATTCCACCCTC |
| dpGATA 105 F | -1667 | AGAAAAGTTAGGCTATCGCAGCA |
| dpGATA 105 R |  | ACCTAGGCTCACTGGTCCCC |
| GATA-105b F | -1203 | CAGCCGGGTTTCACTCGTAC |
| GATA-105b R |  | GGCCCTTTAAATGTAGCAAAGC |
| GATA-106b F | -694 | GAGCTACGCAATCTGACCGG |
| GATA-106b R |  | CAACCTGAGTAGCAAGGAGCG |
| GATA-106c F | -206 | TGATCGGAAGAGCAACCGTC |
| GATA-106c R |  | AGAGAGGAATCCGAGTGTGACC |
| GATA-107 F | 242 | CTCAACGGTCAGCACCCAG |
| GATA-107 R |  | AGCGGATACTGAGCTTCCATG |
| GATA-107b F | 340 | CATCGATGGTCAAGGCAACC |
| GATA-107b R |  | GATACCTCTGCACCGTAGCCC |
| dpGATA 108 F | 579 | GAGGAAGCAGTGTCGCTGGT |
| dpGATA 108 R |  | CCTGCTACGTGCGCTCTTTT |
| GATA-108b F | 1070 | CCCGGCTACACAGAATTTTCC |
| GATA-108b R |  | GAGAAAAGTCGGCCAGGATTG |
| dpGATA 109 F | 1821 | AATGTCAAATGGTGAGGCGG |
| dpGATA 109 R |  | TTTAAACTCGGAGCGCAAATC |
| dpGATA 110 F | 3335 | GATCCCCTACCGGGTTCG |
| dpGATA 110 R |  | GGTAGAGGTGGCGCTTACCTG |
| dpGATA 111 F | 4142 | TGGGAGTTGATTGGAGGCC |
| dpGATA 111 R |  | GGGAGAGCAGAGGCACACAC |
| dpGATA 112 F | 4931 | AGTGTGGTCTGCTGAAGGTGC |
| dpGATA 112 R |  | CTGTTTCTAGTCCACCGCCC |
| dpGATA 113 F | 6178 | TTAGGAAAATTGGCTGTGTACTGAA |
| dpGATA 113 R |  | CCTTGCGTGTGTTTTTCTCTTGT |
| dpGATA 114 F | 6752 | CTCCCCACAGCCACTAGCC |
| dpGATA 114 R |  | TGGGCCGCTTCCTAGTCAG |
| dpGATA 115 F | 7978 | TGCAGATCTCCTGCCTAAAGG |
| dpGATA 115 R |  | AGTCAGCTCTTTAGCCCCAGC |
| dpGATA 116 F | 8939 | TACCTTTGCAATGCCTGCG |
| dpGATA 116 R |  | CGCTTGGGCTTGATAAGGG |
| dpGATA 117 F | 10077 | ACACCCGCTTCCTTAAAGCA |
| dpGATA 117 R |  | GGATGTGAAAGGTGTTAAGTCGTG |
| dpGATA 118 F | 10924 | GCTACACAGAGACACGGAGACTTTT |
| dpGATA 118 R |  | ACACACCCCATAGCATGCAG |
| dpGATA 119 F | 11881 | AGAGAGCACAAGAGGGAAATGG |
| dpGATA 119 R |  | CATTTGGCCACCTGCCTTT |
| dpGATA 120 F | 12712 | CGTGTTCGCCATTGTAGGAA |
| dpGATA 120 R |  | TCCCTAAACCTGAGTGGTGGA |
| dpGATA 121 F | 14192 | CCTGGCTAATTGGAAATTGCC |
| dpGATA 121 R |  | CCTCCCTGGGAACATTACCA |

| **C. NeuroD4 oligonucleotides** | | |
| --- | --- | --- |
| Name | Position | Sequence (5'-3') |
| dpM3 101 F | -9220 | TGCTTTATACTGCAGAGCATCCA |
| dpM3 101 R |  | TTCTGTAAAGGGCACACTGTTTTC |
| dpM3 102 F | -7994 | GGCTCTCTGAGGACCAGCC |
| dpM3 102 R |  | TGCACATGTTCCCCAATTTG |
| M3-103 F | -7190 | CAGGTGGCAATATCTACCCCA |
| M3-103 R |  | GGTACCAGCTGAGGCCAGG |
| dpM3 104 F | -6567 | GGGAACAGGCAAATTCTACCC |
| dpM3 104 R |  | TCCCAACTTGGAGGTTCATTG |
| dpM3 105b F | -5700 | CCCACATGATGGCTCTCAACT |
| dpM3 105b R |  | GCCCAAGGAGTCCAGAAGAAG |
| dpM3 106 F | -4351 | AGTTTCACAGAGCAGGGAAGGTC |
| dpM3 106 R |  | TGCACCTCAGGGTCTGTCC |
| M3-106 b F | -4700 | TCACAGTTTTAGGAGTGTCATTCCA |
| M3-106 b R |  | TCGGGCAAAATGAGTCACTCT |
| dpM3 107 F | -3417 | CCTTATTACCCCCAATTACATATGGTA |
| dpM3 107 R |  | GACAGGACATCAGACAGCTTATTTTC |
| dpM3 108 F | -2505 | TTCAACCTGTTCCCTTCTATTGC |
| dpM3 108 R |  | AATTTGAGCAGACCCTGAGCC |
| dpM3 109 F | -1745 | GATTGCACAGCCTTTCTGCC |
| dpM3 109 R |  | AACCCCTTGACAGTTGTGGG |
| M3-109 b F | -1415 | TTCCATCTCTACCCTCACCTTTG |
| M3-109 b R |  | GTAGGAGGAAAGGAATGGACCAT |
| dpM3 110 F | -837 | GCCAGAGCTCCAAGGGTTTA |
| dpM3 110 R |  | GGCCTGTTGTGTATTGTTCGTGT |
| M3-110 b F | -237 | AGCAGCCCACACCCTCTAGTT |
| M3-110 b R |  | CCAGGTCCAGCTTTGCCA |
| dpM3 Txn Start F | 16 | TGACCCCGGGAAAGAGGTAC |
| dpM3 Txn Start R |  | GATGCCTTCATGTCAGTGCCT |
| dpM3 111 F | 526 | GGTCTGGGCAAACAGTGGA |
| dpM3 111 R |  | TGACCTCCCAAGATCTCCCTC |
| dpM3 112 F | 1827 | TTCGGGATACCTATAAGATGGCA |
| dpM3 112 R |  | TTCAGAGCAAAACCCAAAACCTA |
| M3-112 b F | 1212 | ACATTAGCCTGTATAACCGTTTTCTGT |
| M3-112 b R |  | CTGATGTTACTTGATTACCTCTTGCTG |
| dpM3 113 F | 2541 | TGGTTATTTCTCACCCTTATGTGG |
| dpM3 113 R |  | GGGTTGATGCATTAAACCCTTG |
| dpM3-113b F | 2818 | CAAGGTGGCATTCAGATAACAACA |
| dpM3-113b R |  | AATCAAATTCTCCCCACAGATGA |
| dpM3 114 F | 3397 | AACCCCAGTTCCTGGTAGGG |
| dpM3 114 R |  | TCTCTGTTCCTCATAACTCTTGCTTG |
| dpM3-114b F | 3736 | TTCCTTGGATAATGATCACTCCTG |
| dpM3-114b R |  | AAAGCCATTTTGTCTTTAGCAATTG |
| M3-114 c F | 3904 | TTACATGTCCATCATTTAAAATACTGCC |
| M3-114 c R |  | GCATACCGTTGAAGGTGATGAGT |
| dpM3 +4400 A F | 4299 | AGCTGCTTACCCAGGAAGCC |
| dpM3 +4400 A R |  | TCATATTAATCAGCAAGGAAATCATGT |
| dpM3 +4400 B F | 4234 | TGGGATTTTCTGACTTGGCC |
| dpM3 +4400 B R |  | GCAGCTTTATTGAAAACACACATTG |
| dpM3-114-115 F | 4110 | CCTGCCTCTGGAAGATCTGTG |
| dpM3-114-115 R |  | CGGGCAAATATCCAGAGGG |
| dpM3-115 F | 4517 | GATTGGAGACCCCAGCTTCA |
| dpM3-115 R |  | CACCTTTGATTATGAGAAAGGGAAGA |
| dpM3-115b F | 5005 | TGTGATATTCATGTGCAAAGGAAA |
| dpM3-115b R |  | CAAATTGCCACTGGCCATTA |
| dpM3 116 F | 5182 | CAGTCCAGCCACAGTGGAACT |
| dpM3 116 R |  | CACATTCCCCACTGGCTTTG |
| dpM3-116b F | 5921 | GCCACGCTCTTCTGGTTACCT |
| dpM3-116b R |  | AGCTCAGGGAACCCCACAG |
| M3-116 c F | 5574 | TCCATTCTTCCCCACATCCA |
| M3-116 c R |  | CCAACCTATATGTTTGGGAGGC |
| dpM3 117 F | 6273 | TGTTCCACCATTACCTCAGCC |
| dpM3 117 R |  | CATCAACCCAGCCACAAAACT |
| M3-117 b F | 6624 | ATCCAGGCATCCTCCTTTGA |
| M3-117 b R |  | GGATAATCACTCTACAAACCACAGACC |
| dpM3 117-118 F | 7110 | TCTGCATCCTAGCCAGCATG |
| dpM3 117-118 R |  | TCCATACTACACCTGTCAGAATTGC |
| dpM3 118 F | 7948 | GGCAATTATTTATATGCTTACCTATTTCC |
| dpM3 118 R |  | TCCAGGCACACATTCTTTTCTTTA |
| dpM3 119 F | 8850 | TGGATGGACAAAGGTCTGAGC |
| dpM3 119 R |  | CATTCCATAAGAGCCCGGTCT |
| dpM3-120b F | 10005 | TTTGATGGTTTGAGGCCACA |
| dpM3-120b R |  | TCTTTGGCACCATTGGAACA |
| M3-120 c F | 9186 | GCAAGGAACTACATCTGGGCC |
| M3-120 c R |  | ACAAATCCCTTCCCTTCAAGTG |
| dpM3 121 F | 10682 | AATGGTGGTTGGCTGCTATCC |
| dpM3 121 R |  | ATTTTTCCAGTGGCTATGTGTGAG |
| M3-121 b F | 11084 | CAGCCAAGTTTCTCTTCTCCAGA |
| M3-121 b R |  | TGCTGTGGAAGATGGATCATG |
| dpM3-122b F | 11311 | TTCTTCACACTGAGAGTTATTTTGTGTC |
| dpM3-122b R |  | CCAAAGGTGCAAAGTAGAGCAGA |
| dpM3-122c F | 11629 | TGCCCCATAGATATAGCCTCAAA |
| dpM3-122c R |  | TCTCCTTTTCTCTCACTCCAAACA |
| M3-122 d F | 11996 | GCTGTTTTCCCTCCCTTTTCC |
| M3-122 d R |  | TTGCCAAGAAAAACACAATGTGTA |
| dpM3 123 F | 12134 | CAGCCAGTGCAGAGGGTTTAC |
| dpM3 123 R |  | GCCTTCCAAATCTTCTGGCA |
| dpM3 124 F | 13245 | ACCTGAGAGAGGAAGATTCTGTTTCT |
| dpM3 124 R |  | CCTAGGGTCTCCCCCATTGT |
| M3-124 b F | 13643 | GAGGGCACTGAGAACAAGGG |
| M3-124 b R |  | TTTGGTTTCTAGACAATCCTGGATTT |
| dpM3 125 F | 14395 | CAACAAAAACCAGGGAGGGTAC |
| dpM3 125 R |  | GTCAAGATGAGGGCTTTTCAATACA |

| **D. Msc ChIP oligonucleotides** | | |
| --- | --- | --- |
| Name | Position | Sequence |
| dpMusc 34 F | -5769 | TCCCCACCATCATTCTTGCT |
| dpMusc 34 R |  | CCCTTAGACGATGCCTGTGAA |
| dpMusc 35 F | -4827 | CAAAGGCTGACTTGAACCTGG |
| dpMusc 35 R |  | CACAGGCTTACGTCCTTTTTGG |
| dpMusc 36 F | -3953 | TGGACACGGACCTGGACTACT |
| dpMusc 36 R |  | GGCTACGTAAAAGACACCGCA |
| dpMusc 37 F | -2709 | CCTACCTGTATCCAGCCTGGAA |
| dpMusc 37 R |  | AGTCAAAGTCTTGCTATATATCCCTGGT |
| dpMusc 38 F | -2236 | GTGAAGAGGAACAAATGCTGGATA |
| dpMusc 38 R |  | TCTGCGTTCCATAGTAGTGAGCAG |
| dpMusc 39 F | -1379 | TGGGAGGTATCGCCTTGAGA |
| dpMusc 39 R |  | TCATAGCAGGTGGCTGGCA |
| dpMusc 40 F | -391 | CTGGGAGACAAGCCGCACT |
| dpMusc 40 R |  | CATTTTCACCTGCAGCTGGTC |
| dpMusc 41 F | 1065 | CAAACAAAAACTCAGTTGCCCA |
| dpMusc 41 R |  | GAAAGCAGCACTGGCGATTC |
| dpMusc 42 F | 1638 | TTCAGACGTGGCCATTCGT |
| dpMusc 42 R |  | CCTGTTGGCTGCAGAAACG |
| dpMusc 43 F | 2956 | AAGTCTGGTAAAGGTGAAACAATTGG |
| dpMusc 43 R |  | AGATGTGTTTGTATCCCACCCC |
| dpMusc 44 F | 4000 | CAAAAGGGAACCCAAAATGACTA |
| dpMusc 44 R |  | TTCAGACCTCACACCTGAATTACC |
| dpMusc 45 F | 4901 | TGAACTTAAAAAATAGTGTGCAGGCT |
| dpMusc 45 R |  | CCTCCTTTCAGTTGAGTTCAGCTT |
| dpMusc 46 F | 5801 | GAGTCACCCAGGCGGTACTG |
| dpMusc 46 R |  | TCACACTGCCAAGTCTCAAATGTT |
| dpMusc 47 F | 7116 | TGCTTATGTAGTCAGTAACACTGGAAAA |
| dpMusc 47 R |  | CCTAAGGCTTGCCCCTGC |
| dpMusc 48 F | 7992 | TTAAATTGCTGAATCTCTAGGGCA |
| dpMusc 48 R |  | TCATAGTGGCAAAGAAAGGACAACT |
| dpMusc 49 F | 9225 | AAAACAATTAGCACGGGAGCTC |
| dpMusc 49 R |  | AGCAATGATCTCACAGGCAGAG |
| dpMusc 50 F | 9912 | AGCTGTGCTCCACACTGTGC |
| dpMusc 50 R |  | TCCAGTCACATGCTGATGGC |
| Musc-32b F | -7169 | CACCACTGCCTGGCTGCTA |
| Musc-32b R |  | TCTTCATAAAGTTTCATGGATGCC |
| Musc-32c F | -7496 | AGCTTGATTGGCTTTCACACCT |
| Musc-32c R |  | TGGTAAGAGAGAAGAGAGGGACAGTC |
| Musc-32d F | -7894 | TTGACCCAGTCTCAGGAAAACA |
| Musc-32d R |  | GGAAGGTACCATGGAGAGGCA |
| Musc-33b F | -6184 | TGTTTCCAGACAACACAGGCA |
| Musc-33b R |  | GTCACTACCCAACCTACCTTTCACTAT |
| Musc-34b F | -5366 | TGTCTTACACAAAGCCCAGGC |
| Musc-34b R |  | GCGTCGCAGCACCCAG |
| Musc-36b F | -3387 | CCCCCTTTATGTCCAGTTTGG |
| Musc-36b R |  | CCCTCCCCCCACTACAAAA |
| Musc-38b F | -1960 | CTGGGAGGAGAGGAGAAGGAG |
| Musc-38b R |  | AGCGCTGAGGATTAAGGCTG |
| Musc-39b F | -847 | TTGGCAAGGCAACTGTGCT |
| Musc-39b R |  | GACTCAGACCCAACGCTTTGA |
| Musc-40b F | 565 | CGTGCTGAGCAAAGCCTTCT |
| Musc-40b R |  | TTGGAAAGCTTGGTGTCGG |
| Musc-42b F | 2355 | CCACCCACACTGTCCCCTAT |
| Musc-42b R |  | CTATGCTGTGGCAGGGTGAGA |
| Musc-42c F | 2795 | TCGCTATGTAAGCGCAGCC |
| Musc-42c R |  | GGTAATCCCAGCACTAACGGG |
| Musc-43c F | 3254 | GGGTAAGTCCTCCATCCCCA |
| Musc-43c R |  | CCTATAGCTTTATTCTACCGGCCAG |
